# Deficiency of LIMA1 accelerates bladder cancer progression by disrupting PINK1/Parkin-mediated mitophagy

**DOI:** 10.1038/s41420-026-03136-5

**Published:** 2026-06-16

**Authors:** Shuchen Zhao, Shuang Xu, Kai Cao, Yunfeng Shi, Tinchun Wu, Chengyue Wang, Shihui Li, Zhong Lv

**Affiliations:** 1 Department of Urology, Changzhou Medical Center, Changzhou Wujin People’s Hospital, Nanjing Medical University, Changzhou, 213003 China; 2 Department of Urology, Wujin Hospital Affiliated with Jiangsu University, Changzhou, 213003 China

**Keywords:** Bladder cancer, Mitophagy

## Abstract

The LIM domain and actin-binding protein 1 (LIMA1), as a cytoskeletal-associated tumor suppressor, has not yet been clearly characterized in bladder cancer (BLCA). This study found that the cytoskeletal protein LIMA1 plays a key tumor-suppressing role in BLCA. Clinical analysis revealed that LIMA1 is significantly downregulated in tumor tissues and serum, with its low expression positively correlated with clinical stage, pathological grade, and recurrence risk, and predictive of poor prognosis. Functionally, LIMA1 knockout promotes tumor cell proliferation, migration, and invasion, and increases tumor volume by 1.8-fold in a mouse subcutaneous xenograft model. Mechanistically, database prediction and molecular docking confirmed that LIMA1 directly binds to PINK1, enhancing mitochondrial autophagy by activating the PINK1-Parkin pathway. The mitochondrial autophagy inducer uric acid A reverses the malignant phenotype caused by LIMA1 deficiency. In summary, this study reveals the inhibitory role of the LIMA1/PINK1/mitochondrial autophagy pathway in BLCA progression, providing a theoretical basis for novel therapeutic strategies targeting this pathway.

**Figure:**
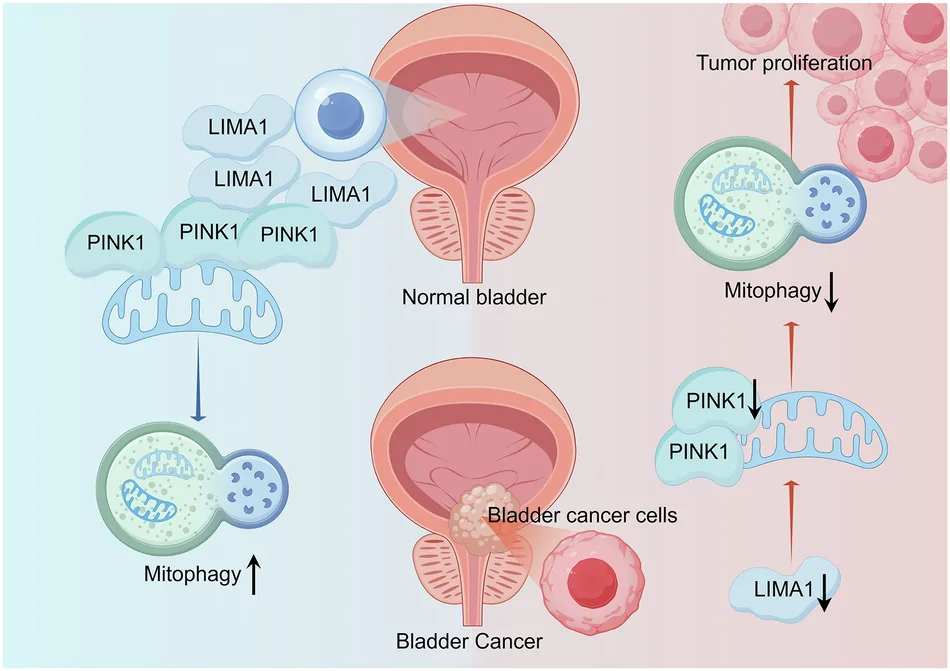

## Introduction

Bladder cancer (BLCA) is one of the most common malignant tumors of the urinary system worldwide, with its incidence increasing alongside population ageing [1]. BLCA is primarily classified into non-muscle-invasive bladder cancer (NMIBC) and muscle-invasive bladder cancer (MIBC). NMIBC has a better prognosis but is prone to recurrence, while MIBC often involves metastasis and has a poor prognosis [2]. Currently, cystoscopic biopsy is the ‘gold standard’ for diagnosing BLCA, but it is an invasive procedure [3]. Therefore, gaining a deeper understanding of the biological mechanisms underlying the development of BLCA and identifying therapeutic targets is crucial for developing new diagnostic and treatment strategies and improving patient outcomes.

LIM domain-containing actin-binding protein 1 (LIMA1), also known as epithelial protein lost in tumors (EPLIN), was initially identified as a novel cytoskeletal protein involved in cell-cell junctions [4–6]. LIMA1 functions as a tumor suppressor in prostate cancer, breast cancer, ovarian cancer, and oesophageal cancer [7–11]. Given its role in maintaining cellular structural integrity and inhibiting metastasis in other urological and epithelial cancers, it is plausible that LIMA1 may play a similarly critical role in BLCA. However, the biological functions and regulatory mechanisms of LIMA1 in BLCA have yet to be investigated.

Furthermore, the metabolic and homeostatic shifts that drive cancer progression often involve the remodeling of intracellular organelles. Mitophagy is a form of selective autophagy that removes damaged or dysfunctional mitochondria, playing a crucial role in maintaining cellular homeostasis [12, 13]. Increasing evidence suggests that mitophagy is closely associated with tumor initiation and progression [14–16]. Notably, our previous study demonstrated that LIMA1 could modulate mitophagy to influence hepatic stellate cell activation, suggesting a novel crosstalk between this cytoskeletal protein and the autophagy machinery [17]. Nevertheless, whether LIMA1 exerts a similar regulatory effect on mitophagy to influence BLCA progression has not been explored, which is the primary focus of the current study.

In this study, we demonstrated that the LIMA1/PINK1-Parkin/mitophagy axis is a novel mechanism in BLCA progression. We found that LIMA1 expression was downregulated in the serum and tumor tissues of BLCA patients, and its expression levels were significantly associated with clinical stage, pathological grade, lymph node metastasis, tumor recurrence, and prognosis. Crucially, our functional and mechanistic analyses revealed that LIMA1 inhibits BLCA proliferation, invasion, and migration by promoting PINK1/Parkin-mediated mitophagy. This study provides new insights into the biological mechanisms underlying the occurrence and development of BLCA.

## Results

### LIMA1 is downregulated in BLCA and associated with poor prognosis

**Fig. 1:**
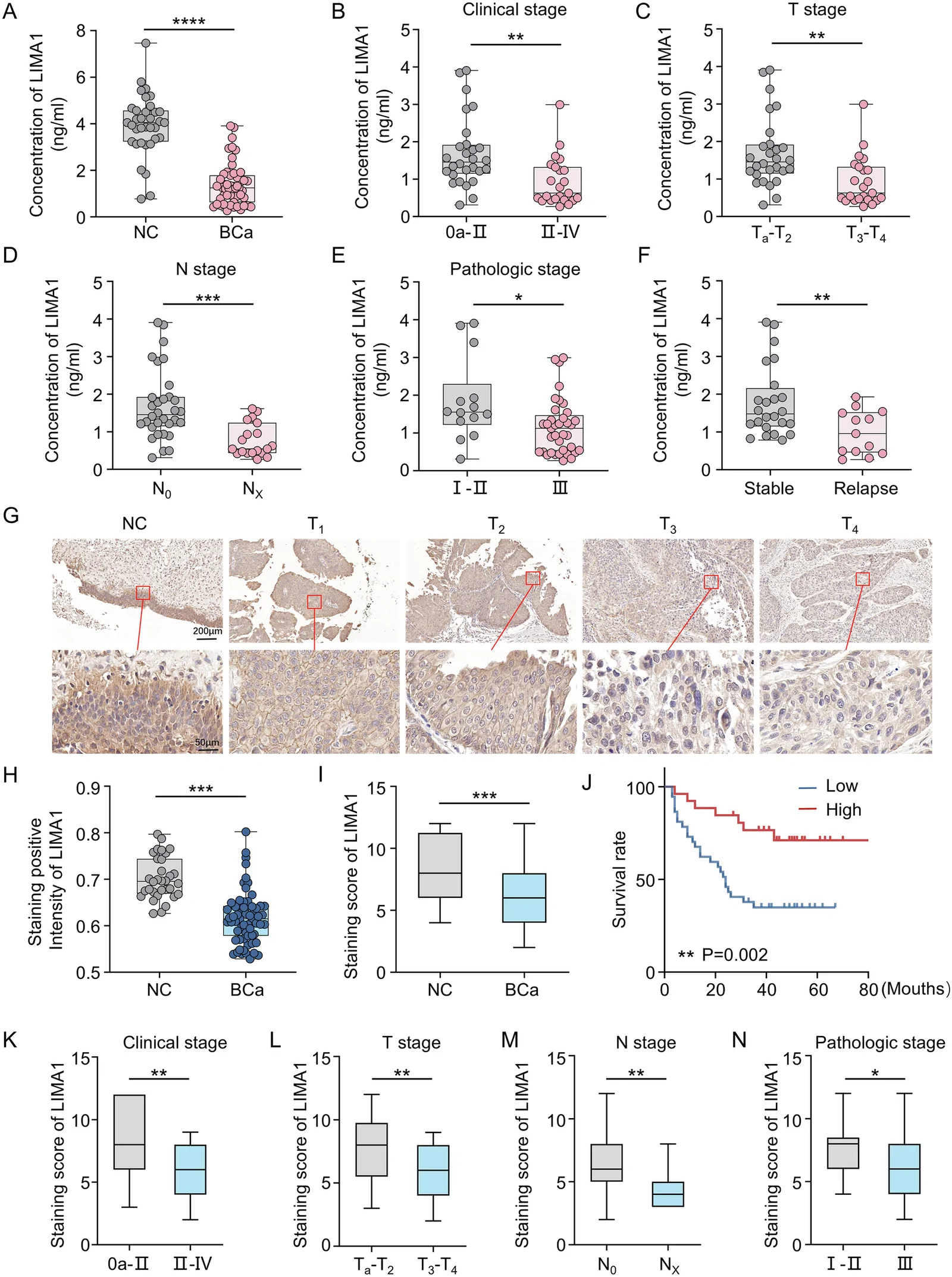
LIMA1 is downregulated in bladder cancer patients. **A** The concentration of LIMA1 was compared by ELISA analysis in serum extracted from healthy controls and BLCA patients. BLCA patients: *n* = 51, healthy controls: *n* = 35. The concentration of LIMA1 by ELISA in 51 BLCA patients, stratified by clinical stage **B**, tumor stage **C**, node stage **D**, pathologic stage **E**, and recurrence **F** from our cohort. Clinical stage 0a-Ⅱ: *n* = 28, Ⅲ-Ⅳ: *n* = 23; Tumor stage Ta-T_2_: *n* = 28, T_3_-T_4_: *n* = 23; Node stage N_0_: *n* = 32, N_x_: *n* = 19; Pathologic stage Ⅰ-Ⅱ: *n* = 14, Ⅲ: *n* = 37. **G** Representative IHC showed that LIMA1 were detected in different T stages from the tissue samples of BLCA patients, scale bar: 200 μm, 50 μm (enlarged). **H** The staining positive intensity of LIMA1 was compared by IHC analysis in bladder cancer sample and corresponding non-tumor normal tissues. Bladder cancer sample, *n* = 63; corresponding non-tumor normal tissues, *n* = 32. **I** The staining score of LIMA1 was compared by IHC analysis in BLCA sample and corresponding non-tumor normal tissues. **J** Kaplan-Meier survival plot depicting cumulative overall survival of 63 BLCA patients, stratified by LIMA1 protein levels using IHC staining scores. The staining score of LIMA1 by IHC in 63 BLCA sample, stratified by clinical stage **K**, tumor stage **L**, node stage **M** and pathologic stage **N** from our cohort. Clinical stage 0a-Ⅱ: *n* = 27, Ⅲ-Ⅳ: *n* = 36; Tumor stage Ta-T_2_: *n* = 30, T_3_-T_4_: *n* = 33; Node stage N_0_: *n* = 53, N_x_: *n* = 10; Pathologic stage Ⅰ-Ⅱ: *n* = 17, Ⅲ: *n* = 46. Data were shown as mean ± SD. *P < 0.05; **P < 0.01; ***P < 0.001; ****P < 0.0001.

To determine the expression profile of LIMA1 in BLCA, we first evaluated serum LIMA1 expression in 51 patients diagnosed with BLCA and 35 healthy individuals. ELISA results revealed that serum LIMA1 concentrations were significantly lower in BLCA patients compared to non-cancerous controls (Fig. 1A). Regarding clinical progression, serum LIMA1 levels were significantly decreased in patients with advanced clinical stage, T stage, N stage, and pathologic stage (all *P* < 0.05, with N stage *P* < 0.001; Fig. 1B–E). When patients were further categorized into high and low LIMA1 groups based on the median serum concentration, low LIMA1 expression was strongly associated with advanced T stage (*P* = 0.003), N stage (*P* = 0.0047), M stage (*P* = 0.0052), Clinical stage (*P* = 0.003), and Pathologic stage (*P* = 0.0094) (Supplementary Table 4). Furthermore, patients with lower serum LIMA1 concentrations were more likely to experience recurrence after transurethral resection of bladder tumor (TURBT) (Fig. 1F). Subsequent ELISA results demonstrated that serum LIMA1 levels were associated with clinical and pathological stages (Supplementary Fig. 1A, B). To assess whether serum LIMA1 expression could serve as a diagnostic marker for BLCA progression, receiver operating characteristic (ROC) curves were plotted for BLCA patients (Supplementary Fig. 2A–F and Supplementary Table 5). Area under the curve (AUC) analysis indicated that serum LIMA1 exhibited high diagnostic efficiency for BLCA staging. To further validate the diagnostic value of LIMA1 expression, univariate Cox analysis revealed that poor prognostic factors in BLCA patients included low LIMA1 expression, TNM stage, clinical stage, and pathological grade (Supplementary Table 6). To further confirm LIMA1 expression in BLCA, we performed immunohistochemical staining on a tissue microarray comprising 95 samples (including 63 bladder cancer specimens) and scored LIMA1 staining in tumor tissues. The results demonstrated that LIMA1 expression was significantly lower in bladder cancer tissues compared to non-tumor normal tissues (Fig. 1G–I). Based on the median LIMA1 expression level, cancer samples were divided into two groups, with low LIMA1 expression strongly associated with poor patient prognosis (Fig. 1J). Similarly, analysis of staining scores confirmed that LIMA1 expression declined in advanced clinical, T, and N stages (*P* < 0.01) as well as pathologic stage (*P* < 0.05) (Fig. 1K–N). This negative correlation was further supported by Kruskal-Wallis analysis, showing a significant association between staining scores and clinical stage (Supplementary Fig. 1C, D). Categorical analysis demonstrated that low LIMA1 staining scores were significantly associated with advanced T stage (*P* = 0.0313), N stage (*P* = 0.0039) and clinical stage (*P* = 0.012) (Supplementary Table 7). Subsequently, ROC curves for BLCA patients were generated using LIMA1 staining scores (Supplementary Fig. 3A–F and Supplementary Table 8), demonstrating that LIMA1 staining scores also had diagnostic efficacy for BLCA staging. Univariate Cox analysis identified low LIMA1 expression, clinical stage, and pathological grade as risk factors for poor prognosis in BLCA patients (Supplementary Table 9). In summary, these findings indicate that LIMA1 is downregulated in BLCA patients and is closely associated with poor prognosis, suggesting its potential as a diagnostic biomarker for BLCA.

### LIMA1 inhibits proliferation, migration and invasion in BLCA

**Fig. 2:**
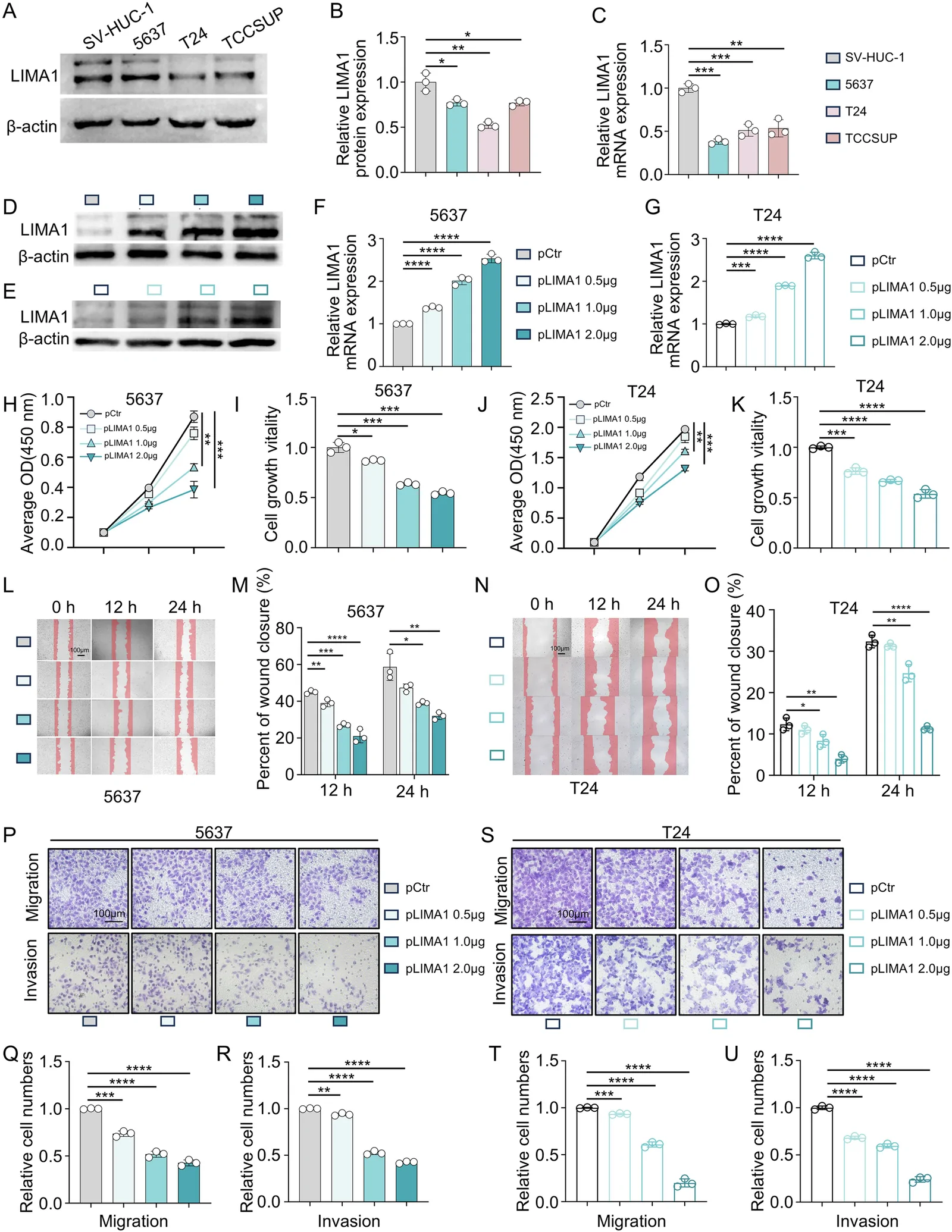
Overexpression of LIMA1 inhibits tumorigenesis of T24 and 5637 cells. **A, B** Expression of LIMA1 was assessed by Western blot analysis in protein extracted from bladder cancer and normal cells. **C** The relative expression of LIMA1 was compared by qRT-PCR analysis in mRNA extracted from bladder cancer and normal cells. **D–G** The overexpression efficiency of LIMA1 in 5637 **D, F** and T24 **E, G** cells were detected by WB and qRT-PCR. **H–K** The overexpression of LIMA1 markedly inhibits proliferation of 5637 **H, I** and T24 **J, K** cells. **L–O** The overexpression of LIMA1 significantly reduces the migration capacity of 5637 **L, M** and T24 **N, O** cells by wound healing assay. **P–R** Transwell assays displayed the migration and invasion of 5637 cells after overexpression of LIMA1. **S–U** Transwell assays displayed the migration and invasion of T24 cells after overexpression of LIMA1. All data were expressed as the means ± SD of at least 3 independent experiments, *P < 0.05; **P < 0.01; ***P < 0.001; ****P < 0.0001.

**Fig. 3:**
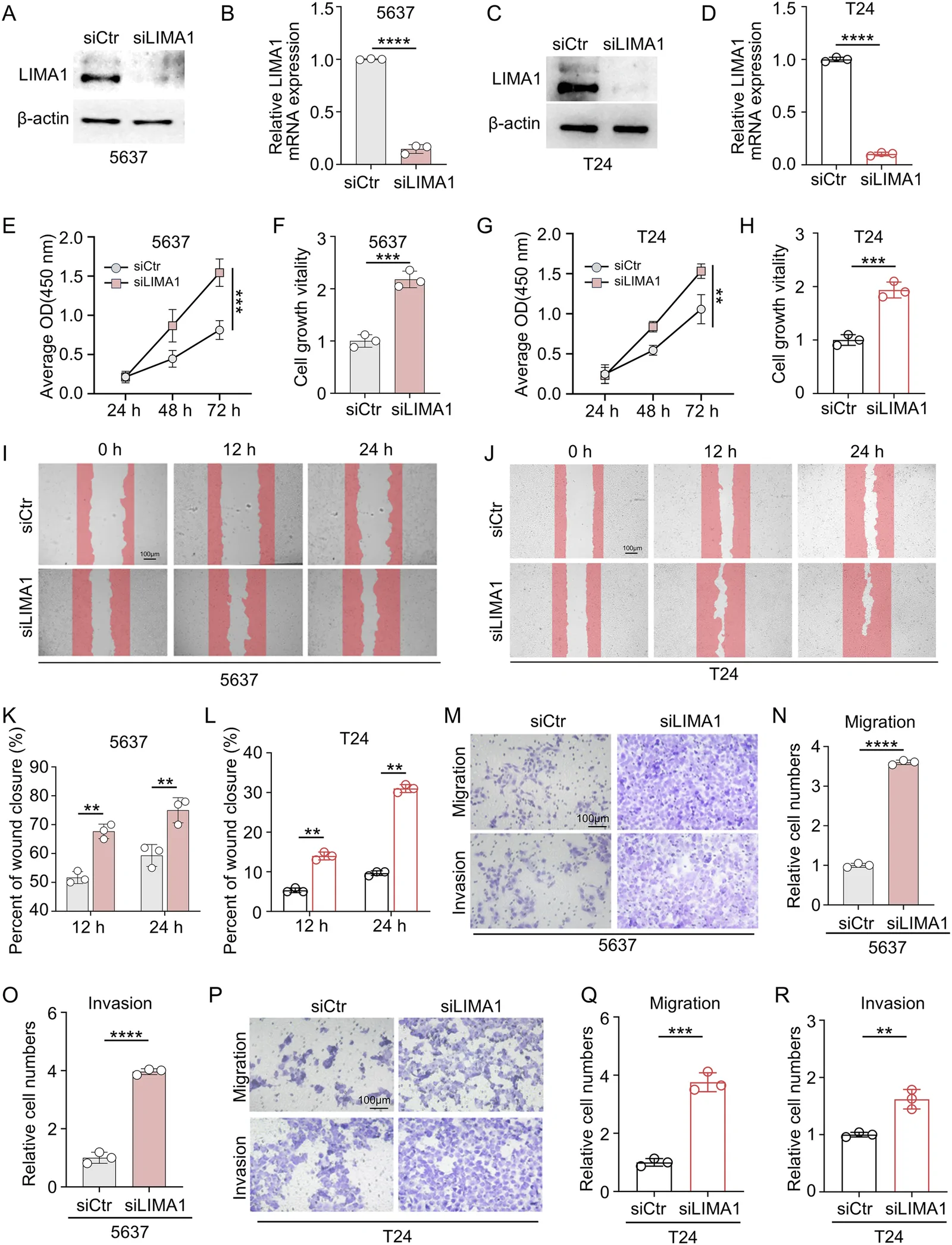
Knockdown of LIMA1 promotes tumorigenesis of T24 and 5637 cells. **A–D** The knockdown efficiency of LIMA1 in 5637 **A, B** and T24 **C, D** cells were detected by WB and qRT-PCR. The knockdown of LIMA1 markedly promotes proliferation of 5637 **E, F** and T24 **G, H** cells. The knockdown of LIMA1 significantly increases the migration capacity of 5637 **I, K** and T24 **J, L** cells by wound healing assay. **M**–**O** Transwell assays displayed the migration and invasion of 5637 cells after knockdown of LIMA1. **P–R** Transwell assays displayed the migration and invasion of T24 cells after knockdown of LIMA1. All data were expressed as the means ± SD of at least 3 independent experiments, *P < 0.05; **P < 0.01; ***P < 0.001; ****P < 0.0001.

To better elucidate the role of LIMA1 in bladder cancer cells, we examined its expression across BLCA cell lines with varying metastatic potential. Both qRT-PCR and Western blot analyses demonstrated significantly reduced expression of LIMA1 protein and mRNA in three BLCA cell lines (TCCSUP, T24 and 5637) compared to SV-HUC-1 human uroepithelial cells. Notably, the high-grade BLCA cell lines TCCSUP and T24 exhibited even lower LIMA1 expression levels than the low-grade 5637 cells (Fig. 2A, C). To investigate LIMA1 functional role across different pathological grades of BLCA, we selected the low-grade cell line 5637 and the high-grade cell line T24 (which exhibited the lowest endogenous LIMA1 expression among the tested lines) for further characterization. These cells were transfected with either control vectors or LIMA1 expression vectors. The efficiency of LIMA1 overexpression was confirmed by Western blot and qRT-PCR (Fig. 2D–G). CCK-8 assays revealed that LIMA1 overexpression significantly impaired the proliferative capacity of both T24 and 5637 cells (Fig. 2H–K). Subsequent evaluation of tumor cell migration and invasion was performed using Transwell and wound-healing assays. The results demonstrated that LIMA1 overexpression markedly delayed wound closure, a key indicator of reduced migratory capacity, in both cell lines (Fig. 2L–O). Furthermore, Transwell assays (with or without Matrigel coating) revealed that LIMA1 overexpression resulted in reduced migratory and invasive capabilities in T24 and 5637 cells (Fig. 2P–U). Conversely, siRNA-mediated knockdown of LIMA1 in T24 and 5637 cells (Fig. 3A–D) resulted in enhanced proliferative capacity, increased migration and greater invasive potential (Fig. 3E–R). These findings collectively demonstrate that LIMA1 exerts tumor-suppressive effects in BLCA by inhibiting cellular proliferation, migration and invasion.

### Knock-down of LIMA1 promoted BLCA progression by downregulating mitophagy

**Fig. 4:**
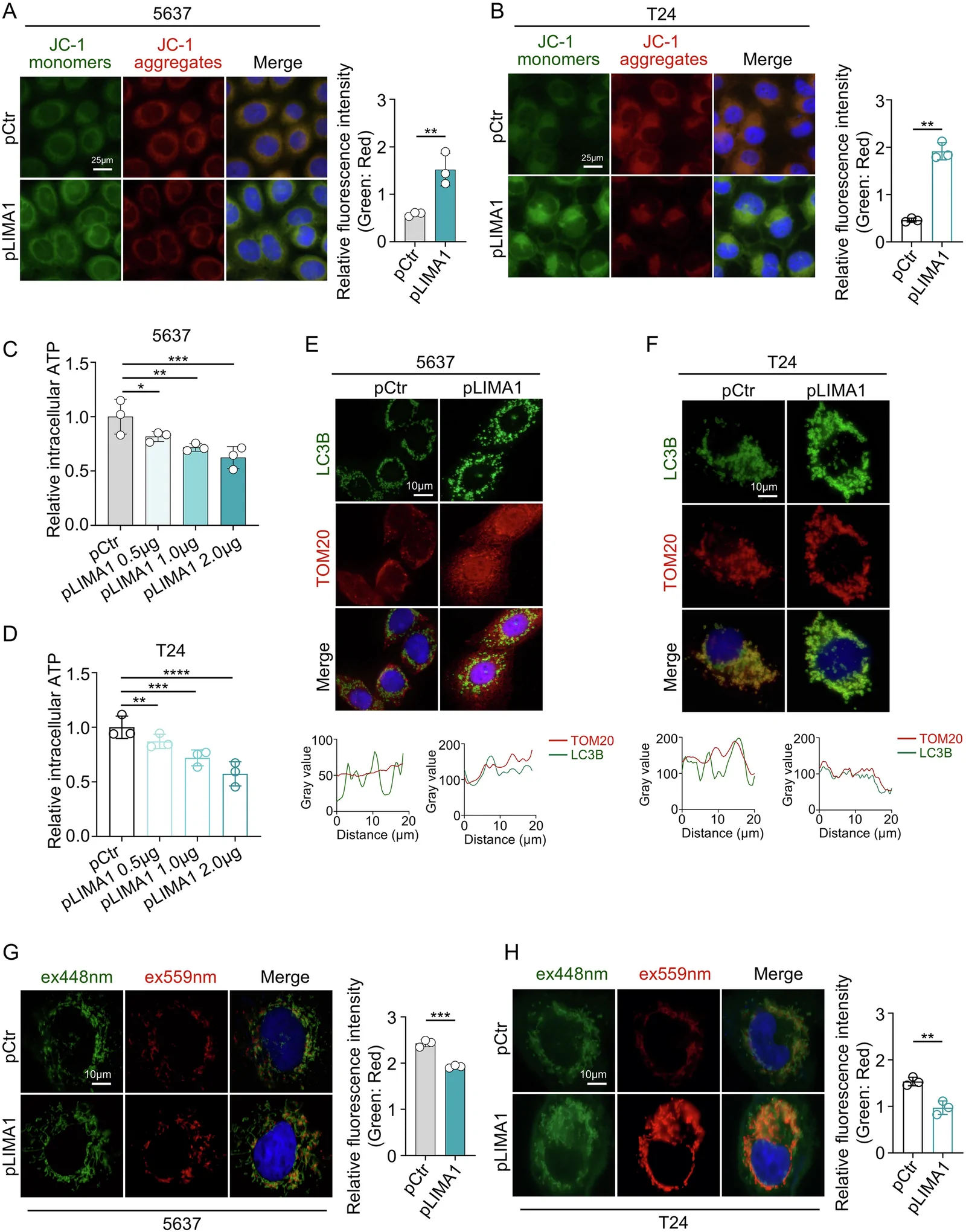
Effects of overexpression of LIMA1 on the mitophagy of T24 and 5637 cells. **A, B**. JC-1 showing the mitochondrial membrane potential in 5637 **A** and T24 **B** cells treated with overexpression of LIMA1. Scale bar = 25 μm. **C, D** Levels of ATP in 5637 **C** and T24 **D** cells after overexpression of LIMA1 by an ATP determination kit. **E, F** Immunofluorescence staining showing LC3B (green) and TOM20 (red) in 5637 **E** and T24 **F** cells treated with overexpression of LIMA1. Scale bar = 10 µm. **G, H** The overexpression of LIMA1 treated 5637 **G** and T24 **H** cells were transfected with mitochondrially targeted mKeima and excitation at 550 nm (red) and 438 nm (green) by microscopy. Scale bar = 10 µm. All data were expressed as the means ± SD of at least 3 independent experiments, **P < 0.01; ***P < 0.001; ****P < 0.0001.

Previous studies have established that LIMA1 is essential for normal mitochondrial function in a cell-autonomous manner and plays a crucial role in solid tumor growth [18, 19]. We therefore hypothesized that LIMA1 might be involved in mitophagy regulation within bladder cancer cells. To investigate LIMA1 impact on mitophagy, we first assessed mitochondrial membrane potential in T24 and 5637 cells overexpressing LIMA1. Compared to control cells, LIMA1 overexpression induced mitochondrial membrane depolarisation (Fig. 4A, B). Since mitochondrial membrane depolarisation is a hallmark of mitochondrial damage and serves as a critical trigger for the initiation of mitophagy, we further examined whether LIMA1 promotes this process. As mitochondria serve as the primary site for aerobic respiration and ATP generation through oxidative phosphorylation, we measured ATP production in both cell lines following LIMA1 overexpression. Notably, LIMA1-overexpressing cells exhibited reduced ATP levels compared to controls (Fig. 4C, D). Subsequent examination of mitochondrial marker TOM20 and autophagosome marker LC3B co-localization revealed that LIMA1 significantly increased their co-localization area (Fig. 4E, F). To further validate the role of LIMA1 in mitophagy, we employed the pH-sensitive mt-Keima probe. In the acidic environment of lysosomes, Mito-Keima excitation shifts from 460 nm to 560 nm. The dual-color fluorescence analysis demonstrated increased red mt-Keima signal in LIMA1-overexpressing bladder cancer cells (Fig. 4G, H), indicating enhanced mitophagy within mature autolysosomes.

**Fig. 5:**
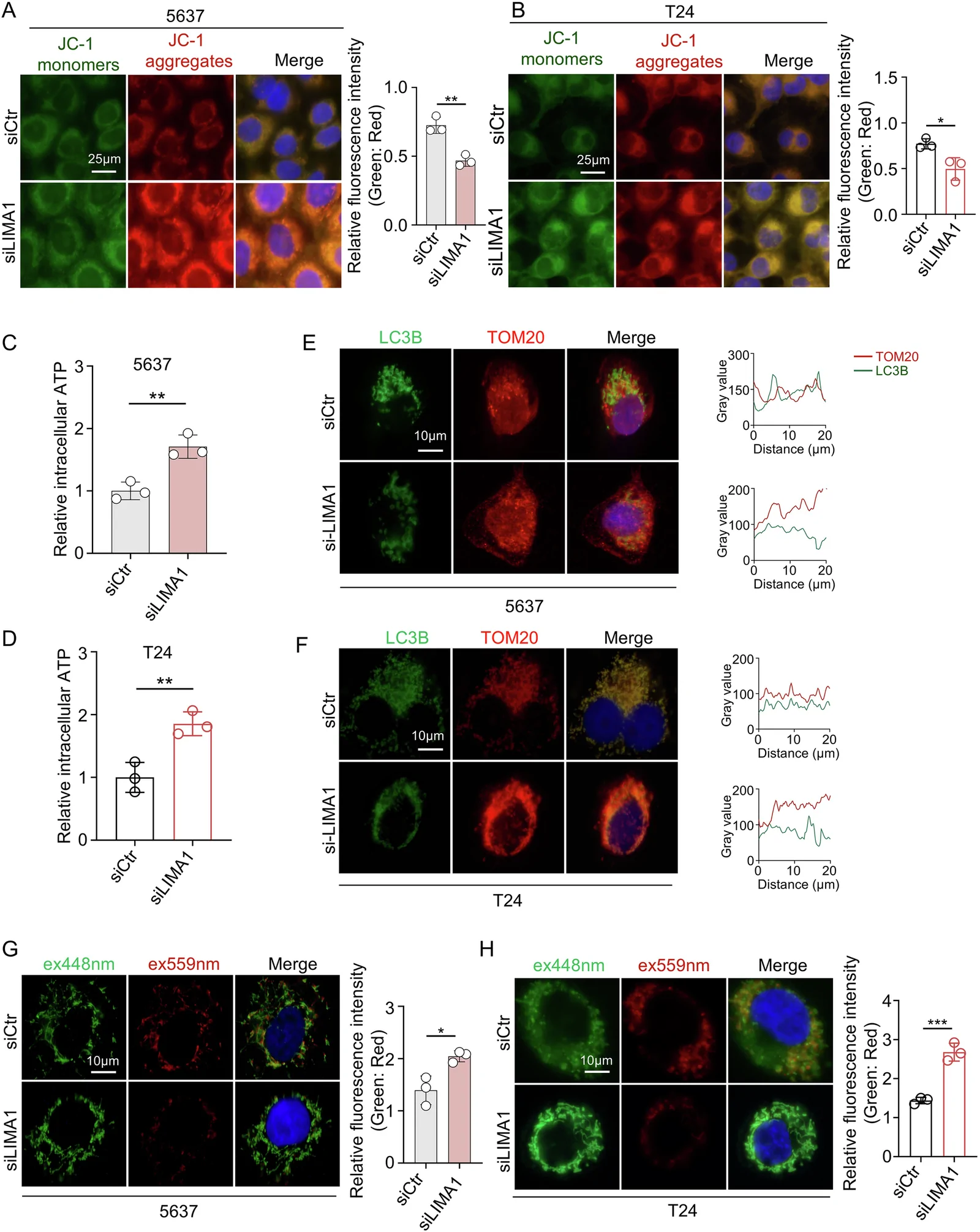
Effects of knockdown LIMA1 on the mitophagy of T24 and 5637 cells. **A, B** JC-1 showing the mitochondrial membrane potential in 5637 **A** and T24 **B** cells treated with knockdown of LIMA1. Scale bar = 25 μm. **C, D** Levels of ATP in 5637 **C** and T24 **D** cells after knockdown of LIMA1 by an ATP determination kit. **E, F** Immunofluorescence staining showing LC3B (green) and TOM20 (red) in 5637 **E** and T24 **F** cells treated with knockdown of LIMA1. Scale bar = 10 µm. **G, H** The knockdown of LIMA1 treated 5637 **G** and T24 **H** cells were transfected with mitochondrially targeted mKeima and excitation at 550 nm (red) and 438 nm (green) by microscopy. Scale bar = 10 µm. All data were expressed as the means ± SD of at least 3 independent experiments, **P < 0.01; ***P < 0.001; ****P < 0.0001.

Conversely, LIMA1 knockdown resulted in elevated mitochondrial membrane potential, increased ATP production, reduced LC3B-TOM20 colocalization, and diminished red lysosomal mt-Keima signal (Fig. 5A–H). These findings collectively demonstrate that LIMA1 promotes mitophagy in bladder cancer cells.

**Fig. 6:**
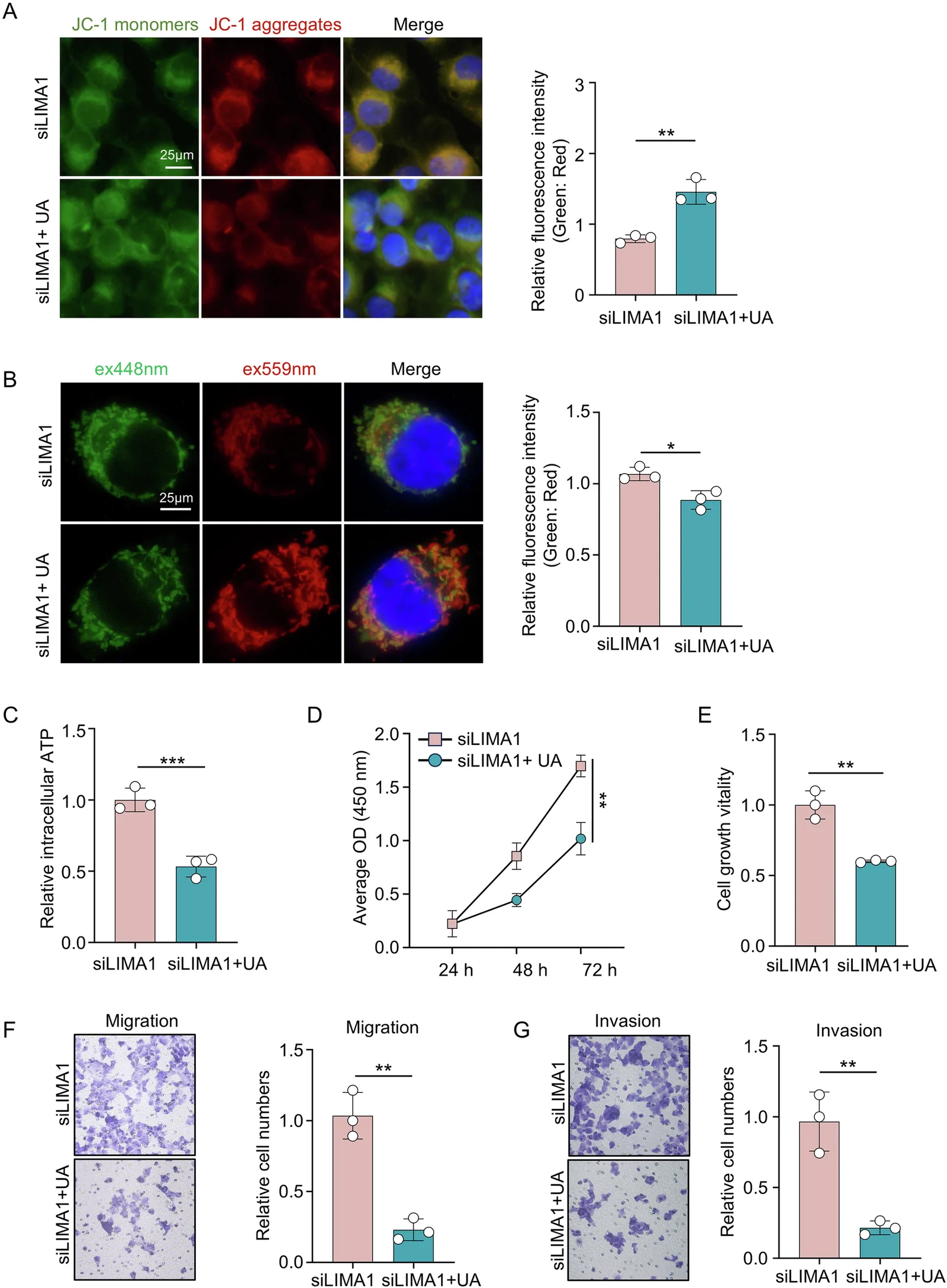
The mitophagy promoter reversed the effect of knockdown of LIMA1 on T24 cell tumorigenesis. **A** JC-1 in siLIMA1-T24 cells treated with Urolithin A. **B** Urolithin A treated siLIMA1-T24 cells were transfected with mitochondrially targeted mKeima. **C** Levels of ATP in siLIMA1-T24 cells treated with Urolithin A. **D, E** The proliferation of siLIMA1-T24 cells treated with Urolithin A. **F** The migration of siLIMA1-T24 cells treated with Urolithin A. **G** The invasion of siLIMA1-T24 cells treated with Urolithin A. All data were expressed as the means ± SD of at least 3 independent experiments, **P < 0.01; ***P < 0.001.

To confirm whether LIMA1 influences BLCA progression through mitophagy regulation, we treated LIMA1-knockdown BLCA cells with urolithin A (UA), a specific mitophagy inducer. UA treatment restored mitochondrial membrane depolarisation in LIMA1-deficient cells (Fig. 6A) and rescued the impaired mitophagy caused by LIMA1 knockdown (Fig. 6B). Importantly, UA supplementation reduced ATP generation (Fig. 6C) and attenuated BLCA cell proliferation, migration and invasion capacities (Fig. 6D–G). In summary, these results demonstrate that LIMA1 plays a critical role in regulating mitophagy, which appears to be closely associated with bladder cancer proliferation and invasion.

### LIMA1 promoted mitophagy through regulation the PINK1/parkin pathway

**Fig. 7:**
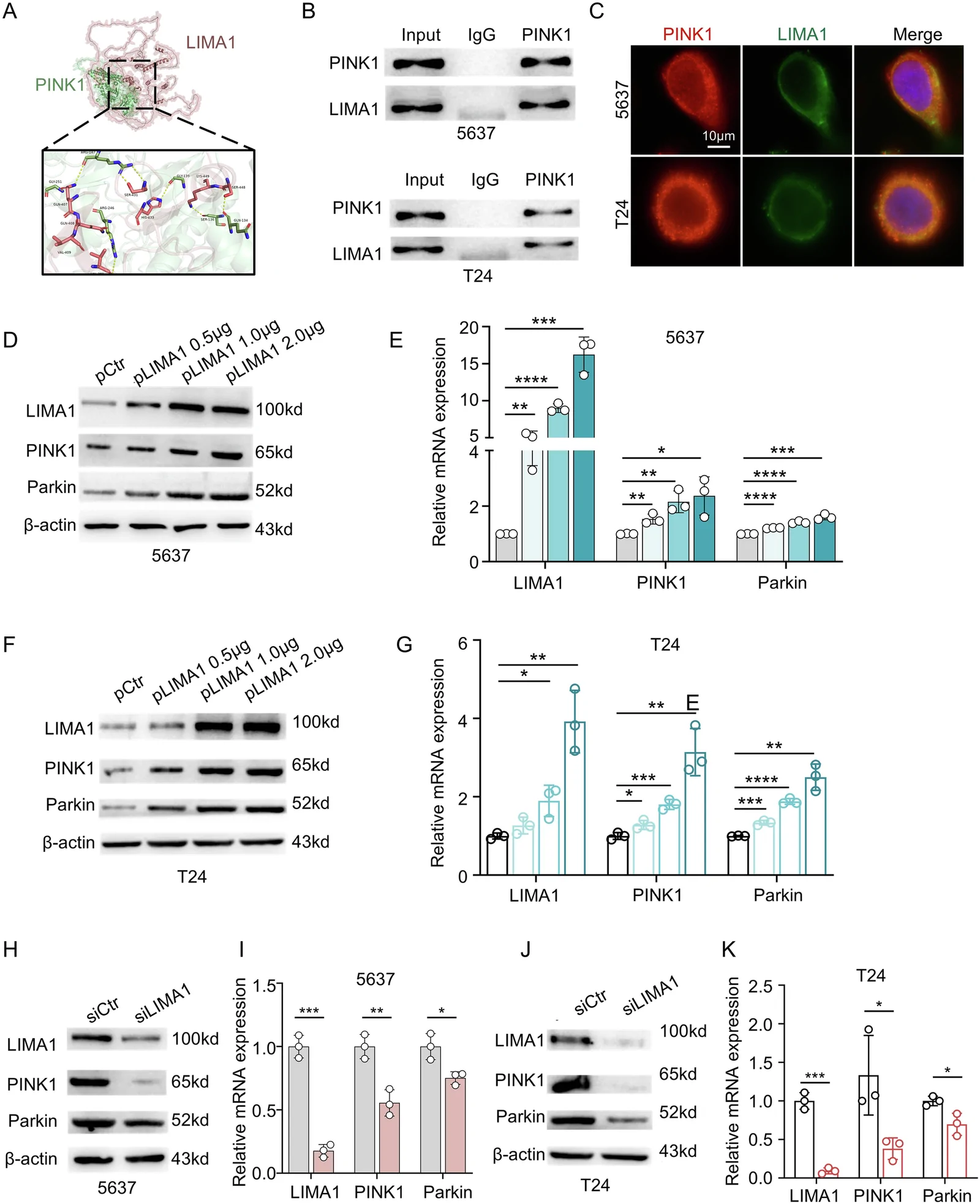
LIMA1 regulated mitophagy through the PINK1/Parkin pathway. **A** Graphical representation of the 3D structure of the LIMA1 and PINK1 interaction model. **B** Co-IP analysis of the endogenous interactions between LIMA1 and PINK1 in 5637 and T24 cells. **C** The co-localization between LIMA1 (green) with PINK1 (red) was analyzed by confocal microscopy in 5637 and T24 cells. Scale bar = 10 μm. **D–G** The expression of PINK1 and Parkin in 5637 **D, E** and T24 **F, G** cells with LIMA1 overexpression by Western blot and qRT-PCR. **H–K** The expression of PINK1 and Parkin in 5637 **H, I** and T24 **J, K** cells with LIMA1 knockdown by qRT-PCR. All data were expressed as the means ± SD of at least 3 independent experiments, **P < 0.01; ***P < 0.001.

Traditionally, the PINK1 (PTEN induced kinase 1) - PRKN (parkin RBR E3 ubiquitin protein ligase) pathway has been thought to be one of the most important molecularly mediated pathways for mitophagy. While our group’s previous study observed that LIMA1 inhibited mitophagy in hepatic stellate cells [17], its functional role in malignant tumors, particularly in BLCA, remained unknown. The LIMA1 protein contains LIM domains, which serve as protein interaction sites enabling LIMA1 to either homodimerise or bind with other proteins. To elucidate the molecular mechanism by which LIMA1 regulates mitophagy in bladder cancer cells, the proteins that interact with LIMA1 and their structures were analyzed using the Protein–protein interaction (PPI) database hitpredict (http://www.hitpredict.org/) and the GRAMM docking server. According to the molecular simulations, multiple residue pairs between LIMA1 and PINK1 capable of forming hydrogen bonds (Fig. 7A).

Subsequently, we conducted co-immunoprecipitation experiments to validate the interaction between LIMA1 and PINK1. Endogenous interaction between LIMA1 and PINK1 was confirmed in both T24 and 5637 cells (Fig. 7B). Furthermore, fluorescence microscopy demonstrated intracellular colocalization of LIMA1 with PINK1 in these cell lines (Fig. 7C).

Western blot analysis showed that overexpression of LIMA1 in 5637 and T24 cells increased intracellular PINK1 and Parkin protein levels in a concentration-dependent manner (Fig. 7D, F). Notably, LIMA1 overexpression enhanced both PINK1 and Parkin mRNA levels (Fig. 7E, G). Conversely, LIMA1 knockdown reduced PINK1 and Parkin expression at both mRNA and protein levels (Fig. 7H–K). Considering that LIMA1 primarily functions as a scaffold protein rather than a transcription factor, these results suggest that LIMA1 likely promotes the PINK1-mediated mitophagy pathway through multiple layers. While the observed changes in mRNA levels may reflect indirect regulatory effects, the significant upregulation of protein levels supports a critical role for LIMA1 in modulating the stability or expression of the PINK1/Parkin complex. In summary, these findings indicate that LIMA1 promotes mitophagy by positively regulating PINK1 and Parkin expression.

### LIMA1 suppressed tumor growth in vivo through enhanced mitophagy

**Fig. 8:**
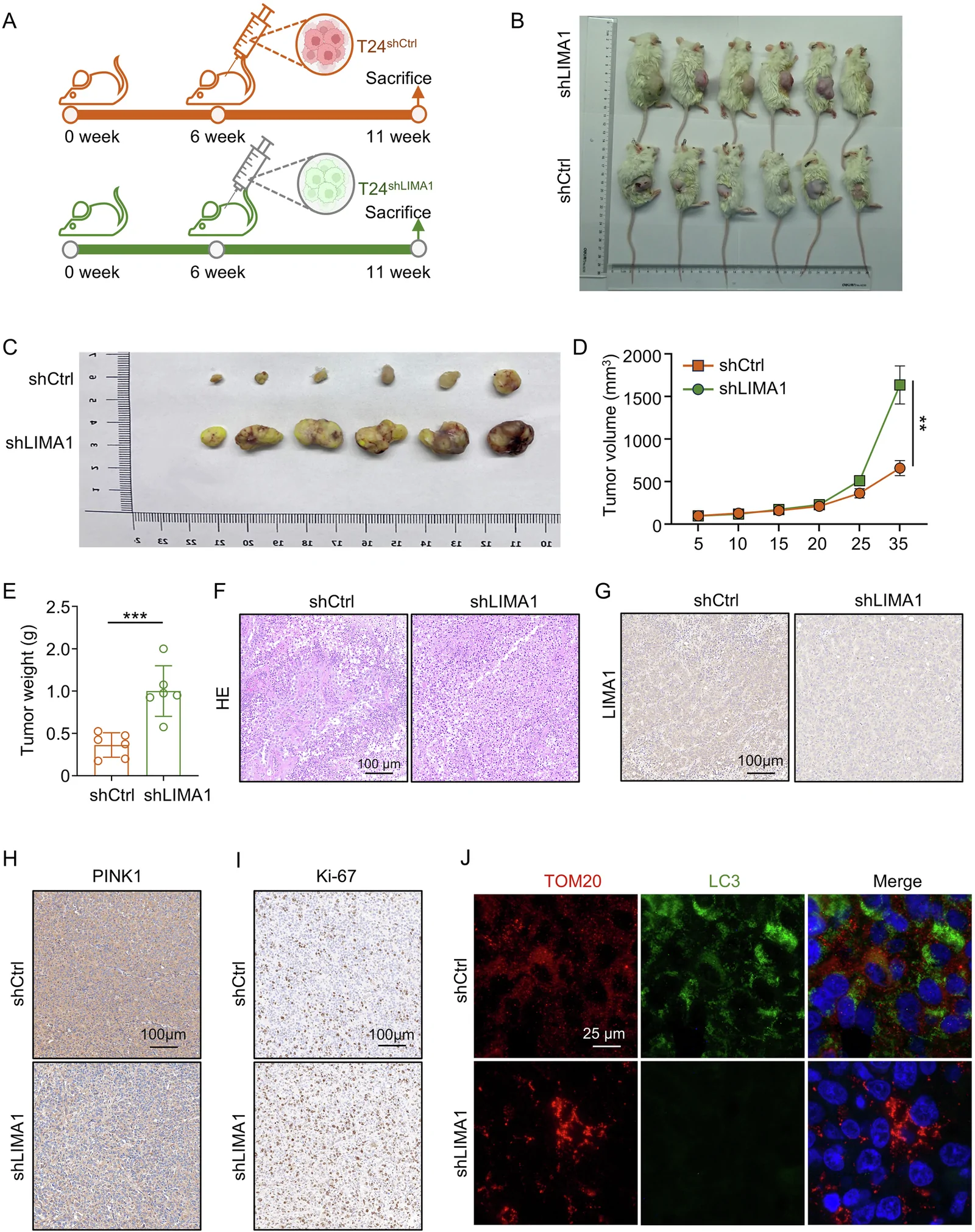
Knockdown of LIMA1 suppressed tumor growth in mice. **A, B** The highly immunodeficient mice grown normally to 6 weeks were injected with shCtrl-T24 or shLIMA1-T24 cells and sacrificed after 7 weeks. **C** The subcutaneous tumors were surgically excised and photographed in two experimental groups (*n* = 6 mice per group). **D** The tumor volume was measured at different time points (*n* = 6 mice per group). **E** The tumor weights of the different groups (*n* = 6 mice per group). **F** H&E staining revealed the tumor tissues in the shLIMA1 group had lesser necrotic areas than that in the shCtrl group. Scale bar:100 μm. **G–I** Representative visual field images of IHC (LIMA1, PINK1, Ki-67) of tumor tissue from two groups. Scale bar: 100 μm. **J** Immunofluorescence staining showing LC3B (green) and TOM20 (red) of tumor tissue from two groups. Scale bar = 25 µm. All data were expressed as the means ± SD of at least 3 independent experiments, **P < 0.01; ***P < 0.001.

To investigate the role of LIMA1 in regulating proliferation and tumorigenesis in vivo, we injected 5 × 10⁶ viable T24 cells transduced with either shCtrl or shLIMA1 into severely immunocompromised mice. The mice were sacrificed at 35 days for tumor weight measurement (Fig. 8A). Compared to mice injected with shCtrl-T24 cells, those receiving shLIMA1-T24 cells exhibited significantly enhanced proliferative capacity and tumorigenicity (Fig. 8B, C). LIMA1 knockdown markedly increased both tumor volume and weight relative to controls (Fig. 8D, E).

H&E staining revealed lesser necrotic areas in tumor tissues from the shLIMA1 group (Fig. 8F). Furthermore, immunohistochemical (IHC) analysis demonstrated significantly reduced LIMA1 and PINK1 levels and elevated Ki67 (a proliferation marker protein) expression in shLIMA1 tumor tissues (Fig. 8G–I). In addition, immunofluorescence staining of TOM20 and LC3 revealed that, compared to the shCtrl group, tumor tissues in the shLIMA1 group exhibited reduced co-localization of TOM20 and LC3, indicating suppressed mitophagy (Fig. 8J). These experimental findings indicate that LIMA1 knockdown enhances tumor formation in vivo by suppressing PINK1-mediated mitophagy.

## Discussion

LIMA1 was widely recognized as a tumor suppressor that was frequently downregulated or lost in various malignancies including lung, oral, prostate, breast, and ovarian cancers, as well as hepatocellular carcinoma, colorectal cancer, neuroblastoma, and gastric cancer [5, 20–25]. However, it has been shown to exhibit tumor-promoting effects in cholangiocarcinoma [26], head and Neck Squamous Cell Carcinoma [27], and pancreatic cancer [28], indicating that the function of LIMA1 displays tissue-specific characteristics across different types of cancer. In recent years, the known functions of LIMA1 have expanded beyond its established roles in regulating cytoskeletal dynamics and cellular motility to encompass cell division, genetic regulation, apical extrusion, angiogenesis, cellular metabolism, and lipid metabolism [29–33]. In the present study, we provided novel evidence supporting LIMA1 was significant role in BLCA. Our findings demonstrated that LIMA1 expression was markedly downregulated in both serum samples from BLCA patients and immunohistochemical staining scores of BLCA tissue microarrays. Elevated LIMA1 expression was shown to reduce BLCA cell viability, proliferation, and migratory capacity. Nevertheless, the precise molecular mechanisms underlying LIMA1 regulation of BLCA progression remain to be fully elucidated and warrant further comprehensive investigation.

LIMA1 has been reported to be involved in intracellular ATP metabolism and mitophagy [17, 34]. Mitophagy is a process by which cells selectively encapsulate and degrade damaged mitochondria through autophagy mechanisms, thereby maintaining mitochondrial and intracellular homeostasis. Therefore, we hypothesized that LIMA1 may regulate mitophagy in BLCA cells, consequently influencing the state of tumor cells. A growing body of research has documented the critical role of mitophagy in the progression of BLCA. Some studies suggested that abnormal mitochondria may alter cellular behavior and promote tumor development, whereas mitophagy can eliminate dysfunctional mitochondria to suppress tumor growth [35]. Conversely, other studies indicated that rapidly proliferating tumor cells exhibit an increased rate of mitochondrial damage. Mitophagy selectively removed dysfunctional mitochondria while preserving healthy ones, ensuring the proper functioning of metabolic networks and meeting the heightened biosynthetic demands of cancer cells [36]. The balance between these opposing roles may determine whether the tumor progresses or undergoes apoptosis. In this study, we demonstrated that LIMA1 promotes mitophagy in BLCA through TOM20 and LC3 fluorescence co-localization analysis, along with mt-Keima fluorescence assays. Furthermore, the proliferative effects of LIMA1 knockdown in bladder cancer could be inhibited by the mitophagy enhancer urolithin A. These findings indicated that LIMA1 influences tumor growth by regulating mitophagy in BLCA cells.

The mechanisms of mitophagy could generally be classified into ubiquitin-dependent and ubiquitin-independent pathways. The key proteins in the ubiquitin-dependent pathway were PINK1 (PTEN-induced putative kinase 1) and Parkin. Under stimuli such as nutrient deprivation, cellular senescence, and elevated reactive oxygen species (ROS) levels, mitochondria undergo depolarisation, leading to a loss of membrane potential [37]. When mitochondria were depolarized, PINK1 failed to undergo normal cleavage and instead accumulated on the outer mitochondrial membrane (OMM), where it phosphorylated attached ubiquitin chains. This triggered the recruitment of Parkin to the OMM and its subsequent phosphorylation and activation. Activated Parkin further ubiquitinated mitochondrial membrane proteins, leading to the recruitment of autophagy receptors to the mitochondria [38]. This study confirmed that LIMA1 increased the expression of PINK1 and Parkin at both the mRNA and protein levels. LIMA1 was initially identified as a cytoskeleton-associated protein [5]. Its central region consists of a cysteine-rich zinc finger LIM domain flanked by two actin-binding domains. This unique protein structure provided interaction sites for binding with other proteins. Since LIMA1 was not a transcription factor, its regulation of PINK1 at the transcriptional level likely occurs indirectly—through binding to PINK1’s transcription factors, thereby modulating PINK1 mRNA expression. Recent studies have shown that the SIRT1-FOXO3-BNIP3 pathway restores PINK1-PRKN-mediated mitophagy in both bladder fibroblasts and HCC cells [39, 40]. Given that this axis has been confirmed as an upstream regulator of mitophagy, we speculate that LIMA1 might influence PINK1 transcription by modulating the SIRT1-FOXO3 axis. While this indirect mechanism requires further validation, it provides a plausible hypothesis for how LIMA1 promotes the PINK1-PRKN mitophagy pathway at the transcriptional level. Li et al. demonstrated that LIMA1 can influence PINK1 protein stability via direct interaction [17]. Our findings align with these observations. Using AutoDock software to predict LIMA1-binding proteins, we found that LIMA1 can physically interact with PINK1. Furthermore, Co-IP and fluorescence co-localization assays confirmed the binding and co-localization of LIMA1 and PINK1 in bladder cancer cells. Thus, these results suggested that LIMA1 may regulate the PINK1-mediated mitophagy pathway through dual mechanisms—both at the transcriptional level and the protein level. Intriguingly, our findings that LIMA1 promotes PINK1-mediated mitophagy in BLCA contrast with a previous study by Li et al., which suggested that LIMA1 inhibited mitophagy during the activation of hepatic stellate cells (HSCs) [17]. This discrepancy highlights the potential tissue-specific and context-dependent roles of LIMA1. While LIMA1-mediated inhibition of mitophagy may favor HSC activation in liver fibrosis [17], our data robustly demonstrate that in BLCA, LIMA1-driven mitophagy acts as a tumor-suppressive mechanism by clearing dysfunctional mitochondria. Such functional duality is consistent with the nature of scaffold proteins, whose biological impact often depends on the specific interacting partners and metabolic demands of the distinct cellular environment.

We further investigated the role of LIMA1 in BLCA using a subcutaneous xenograft model. Knockdown of LIMA1 promoted tumor progression, confirming its function as a tumor suppressor in BLCA. Therapeutically, small-molecule drugs that directly activate LIMA1 protein could hold promise for BLCA treatment. However, no studies have yet reported the development of direct LIMA1 agonists. Therefore, the use of mitophagy enhancers may represent a novel therapeutic strategy in bladder cancer patients with low LIMA1 expression. While these approaches require further validation, they could provide promising new leads for BLCA treatment.

Notably, BLCA patients with lower LIMA1 expression exhibited more advanced clinical staging, higher T-stage, greater nodal involvement (N-stage), and poorer pathological grading, suggesting LIMA1 may be closely associated with disease progression and unfavourable prognosis. Furthermore, we evaluated the potential of LIMA1 as a biomarker through ROC curve analysis. Crucially, patients with high LIMA1 expression demonstrated superior clinical outcomes compared to those with low expression, indicating LIMA1 may serve as a positive prognostic factor in BLCA.

However, our current study has several limitations. For instance, the analysis of clinical characteristics was conducted in a relatively small cohort. Additionally, chemotherapy remains a cornerstone of bladder cancer treatment, yet this study focused solely on the cytological effects of LIMA1 without investigating its potential role in chemoresistance. Secondly, while we have preliminarily established that LIMA1 binds to PINK1 to regulate its expression in bladder cancer, the specific binding sites and underlying mechanisms remain unexplored. Furthermore, the in vitro migration and invasion findings require validation in animal metastasis models. Notably, a key technical limitation is the absence of in vivo mitophagy flux data using the mt-Keima probe. While mt-Keima probe provided compelling evidence for mitophagy in vitro, our in vivo validation relied on complementary markers such as LC3B/TOM20 immunofluorescence due to the complexities of ratiometric imaging in solid tumor microenvironment. Intriguingly, Parkin has been shown to inhibit bladder tumor growth and metastasis through a mitophagy-independent pathway [41], suggesting the existence of additional, yet uncharacterized, PINK1-Parkin-mediated mechanisms by which mitophagy suppresses bladder cancer progression via LIMA1. Consequently, our findings warrant further investigation through expanded cohorts to better elucidate the role of LIMA1 in bladder cancer, particularly its influence on BLCA cell sensitivity to chemotherapeutic agents. In summary, we have identified a novel role for LIMA1 in regulating BLCA progression. Our work demonstrated that LIMA1 deficiency in BLCA cells may impair PINK1/Parkin-mediated mitophagy, thereby promoting tumor proliferation, migration and invasion. Therefore, LIMA1 acted as both a tumor suppressor in BLCA and represented both a potential novel prognostic biomarker and therapeutic target for patients with this malignancy.

## Materials and methods

### Clinical specimens

The study using serum from bladder cancer patients (*n* = 51) and healthy subjects (*n* = 35) was approved by the Institutional Ethics Committee of Wujin Hospital of Public Jiangsu University (approval number: 2024-SR-016). Eligible participants were required to be aged ≥18 years. The exclusion criteria were set as follows: participants with other malignancies, acute or chronic inflammatory diseases, or major systemic disorders. Furthermore, there were no statistically significant differences between the healthy subjects and BLCA patients in terms of age, sex, and smoking history.

The bladder tissue microarray was commercially purchased from SHANGHAI TUFEI BIOTECH CO., LTD. It contained 63 bladder cancer samples and 32 matched adjacent normal tissues obtained from the same cohort of patients between 2007 and 2011.

### Enzyme-linked Immunosorbent Assay (ELISA)

LIMA1 levels in serum from subjects were measured by Human LIM Domain And Actin-Binding Protein 1 (LIMA1) ELISA Kit (abx259562, Abbexa, USA).

### Western blot

RIPA buffer (Beyotime, China, P0013K) was used to lyse samples from different groups at 4 °C. Cell lysates were separated by 10% SDS-PAGE and transferred onto polyvinylidene fluoride membranes. The primary and secondary antibodies used in this study are listed in Supplementary Table 1. Protein bands were visualized using an enhanced chemiluminescence (ECL) solution (Beyotime, China, P0018M) according to the manufacturer’s instructions and quantified using Image J software.

### Quantitative RT–PCR (QRT-PCR)

For qPCR analysis, the total RNAs from cultured cells or tissue were isolated by TRIzol reagent (Invitrogen, USA) according to the manufacturer’s instructions. The concentration and purity of RNA were determined by NanoDrop 2000 spectrophotometer (Thermo Fisher Scientific, USA). Subsequently, 1 μg of total RNA was reverse-transcribed into cDNA using the PrimeScript RT Reagent Kit (Takara Biomedical Technology, Japan). QPCR was performed on ABI Prism 2720 PCR system (Applied Biosystems, USA). The PCR cycling conditions were as follows: initial denaturation at 95 °C for 30 s, followed by 40 cycles of 95 °C for 5 s and 60 °C for 30 s. β-actin was employed as the internal control for data normalization and relative expression levels were calculated using the 2^−ΔΔCt^ method. Primer sequences are listed in Supplementary Table 2.

### Immunohistochemistry

Deparaffinized paraffin sections were immunohistochemically stained for primary antibodies (Supplementary Table 1), using the DAB kit (BOSTER, China, 18E16B22) per the manufacturer’s instruction. The Classifier module in the Halo v3.0.311.314 analysis software was used to distinguish the tumor regions, and the Indica Labs - Area Quantification v2.1.314 module in the Halo v3.0.311.314 analysis software was used to quantify the positive area and positive intensity of the target region of each slice, respectively. The percentage of positive staining was scored as follows: 0 for 0 ~ 5% of cells stained; 1 for 6% ~ 25% of cells stained; 2 for 26% ~ 50% of cells stained; 3 for 51% ~ 75% of cells stained; 4 for more than 75% of cells stained. The staining intensity of the cells was scored as 0 for not stained, 1 for weakly positive, 2 for moderately stained and 3 for strongly positive. The IHC scores (0–12) were the product of the cell positivity rate score and the staining intensity score. If the percentage of stained cells was zero or the staining intensity was negative, the final score was recorded as 0. Both the frequency and intensity were assessed in a double-blind manner.

### Cell culture

The BLCA cell lines (TCCSUP、T24 and 5637) and the human urethral epithelial immortalized cell line (SVHUC-1 cell) were purchased from the Vigen Biotechnology Co.,Ltd. The SV-HUC-1 cell was cultured in F-12 K (Gibco, USA) medium with 10% fetal bovine serum (FBS) (Gibco, USA). The T24 and the 5637 cells were cultured in RPMI-1640 (Gibco, USA) medium supplemented with 10% FBS (Gibco, USA), while the TCCSUP cell line was cultured in basic DMEM (Gibco, USA) supplemented with 10% FBS (Gibco, USA). All cell lines were cultured at 37 °C in a humidified incubator containing 5% CO_2_. The cell lines described above were verified using short tandem repeat (STR) assays. Mycoplasma was not detected in any of the cell lines.

### Cell transfection

To down-regulate or up-regulate the expression of LIMA1, the LIMA1 expression vector and control vector (purchased from Fenghui Biology Co., Ltd.), siLIMA1 and scramble control siRNA were used (purchased from GenePharma Co., Ltd.). The siRNA sequences were listed in Supplementary Table 3. Briefly, cells were seeded 24 h prior to transfection. Specifically, plasmids were transfected using LipoFiterTM3 (Hanbio Biotechnology Co., Ltd.), while siRNAs were transfected using Lipofectamine 2000 (Invitrogen, USA) according to the respective manufacturer’s protocols. Cells were harvested 24 h post-transfection for RT-qPCR analysis and 48 h post-transfection for Western blotting. Functional assays were initiated 24–48 h after transfection to ensure optimal protein knockdown or overexpression.

### Cell counting Kit-8 (CCK-8) assay

5637 and T24 cells (10^4^ cells per well) were cultured overnight in 96-well plates with different treatments. After that, cell viability was evaluated using a CCK-8 kit (Beyotime, China, BS350A). The absorbance at 450 nm was measured using a microplate reader.

### Wound healing assay

The different groups of cells were placed into plates, and when the cells reached 100% confluence, we scratched the cells with a pipette tip. The cells were incubated in a medium without FBS for 24 h and then photographed with a microscope.

### Cell migration and invasion assay

The migration and invasion assays were performed by suspending the cells in a medium lacking serum and plating 2–10 × 10^4^ cells/200 μl into a 24-well plate containing a polycarbonate pore filter (CORNING, China, 3422) with or without Matrigel (CORNING, China, 354234) 24 h after transfection. The lower chambers were incubated with 500 μl of the cell culture medium with 20% FBS at 37°C for 48 h. Swabs were then used to remove the cells from the chambers. Paraformaldehyde was applied for fixation, and crystal violet was used to stain the cells that migrated or invaded outside their chamber. The stained cells were visualized and photographed by light microscopy.

### ATP assay

The ATP assay was performed according to the instruction of ATP assay kit (Beyotime, China, S0027). In short, the cells were harvested and lysed with a lysis buffer. 20 µl lysate was mixed with 100 µl of luciferase reagent, which catalyzed the light production from ATP and luciferin. Luminance was measured by a monochromatic microplate reader.

### Measurement of mitochondrial membrane potential

The mitochondrial membrane potential was measured using the JC1 Mitochondrial Membrane Potential Assay Kit (Beyotime, China, C2003S). Cells were incubated with JC-1 staining solution for 20 min at 37 °C in the dark. Fluorescence images were captured using a fluorescence microscope (Olympus, Tokyo, Japan) in a randomly selected field of view.

### Immunofluorescence

After the cells fixed with 4% paraformaldehyde were permeated with 0.1% Triton X-100, 5% BSA solution was used to block the nonspecific binding sites. Cells were then incubated with the primary antibodies and the secondary antibody (Supplementary Table 1). The fluorescence images were acquired using a fluorescence microscope (Olympus, Tokyo, Japan) in a random field of view.

### Mitochondrial autophagy promoters

To investigate the role of mitophagy in BLCA activation, we conducted experiments using mitophagy promoters Urolithin A (Solarbio, China, IU0200, 50 μM) in co-cultivation with 5637 and T24 cells for 48 h. Subsequently, the cells were used for further analysis.

### Co-immunoprecipitation (Co-IP)

Cells were lysed in an ice bath with lysis buffer for 30 min. Cell lysates were incubated with the anti-LIMA1 antibodies for 2 h. Then protein A/G agarose beads (Beyotime, China, P2197S) were added and the mixtures allowed incubating at 4°C with gentle rocking overnight. Beads were washed with an appropriate amount of lysis buffer and eluted before analysis by Western blot.

### Protein–protein docking

To ensure the accuracy of the docking results, proteins were prepared for docking with Autodock Tools v. 1.5.7. Docking Web Server (GRAMM) was used for protein–protein docking. The resulting protein–protein complex was also manually optimized by removing water and adding polar hydrogen by the AutoDockTools-1.5.7. Finally, the protein–protein interactions were predicted and the protein–protein interaction figure was generated by PyMOL (https://pymol.org).

### Lentiviral transfection

To construct lentivirus-infected T24 cells with knockdown of LIMA1, lentiviral knockdown vectors targeting LIMA1 were purchased from Vigen Biotech (Zhenjiang, China). Lentiviral sequences are as follows: 5′- GTCTCTGAATTGGTCGAGTTT-3′. The negative control vector was used as control. The infection was done at a multiplicity of infection (MOI) of 50% confluency. Twenty hours after transfection, the medium containing virus was replaced with fresh culture medium. Stably transfected cells were selected by adding puromycin (1 μg/mL) into the medium. Finally, these LIMA1-knockdown cells were used in animal experiments. For the in vitro detection of mitophagy, mt-Keima-COX8 lentivirus (purchased from Vigen Biotech) were infected at 70% cell fusion rate. Selecting infected cells with puromycin at an appropriate concentration after 48 h.

### Animal experiments

All animal experimental procedures were approved by the Institutional Animal Management & Use Committee of Jiangsu University (approval number: UJS-IACUC-AP-2024022801). We purchased 12 NOD-Prkdcscid Ⅱ2rgtm201(-/-)/V highly immunodeficient mice (6 weeks old) from VIEWSOLID Bio Technology (Beijing, China) and adaptively fed them in a specific pathogen-free (SPF) facility for 1 week. We constructed two T24 cell lines (negative control vector and shLIMA1 vector). A total of 5 × 10^6^ cells (per mouse) were resuspended in 100 μl PBS: Matrigel (1:1) and subcutaneously injected into the dorsal flank of each mouse, respectively (n = 6 per group). We measured tumor length (L) and width (W) every five days and calculated tumor volume (V) using the formula V = 0.5 × L × W^2^. The mice were sacrificed for tumor weighing and IHC staining. Additionally, mitophagy in the tumor tissues was evaluated by double-labeling immunofluorescence of LC3B and TOM20 as an in vivo validation.

### Statistical analysis

GraphPad 9.5.1 (La Jolla, CA, USA) was used for statistical analysis. All the quantitative data are presented as the mean ± standard deviation (SD). Data distribution was assessed for normality using the Shapiro-Wilk test. For normally distributed data, unpaired multiple t tests (for two groups) and analysis of variance (ANOVA) (for multiple groups) were employed. The statistical tests were two-sided, and values with *P* < 0.05 were considered to indicate statistical significance. For data that did not follow a normal distribution, the Mann-Whitney U test or Kruskal-Wallis test was used accordingly. To determine the association between LIMA1 expression and clinical features, continuous LIMA1 expression levels were compared across groups using the Mann-Whitney U test for dichotomous variables and the Kruskal-Wallis test for multi-categorical variables; meanwhile, patients were dichotomized into high and low LIMA1 expression groups based on the median value, and the Chi-square test or Fisher’s exact test was used to evaluate frequencies across clinical subgroups. The diagnostic capability of biomarkers was assessed by receiver operating characteristic (ROC) curves. The survival curves were obtained by the Kaplan–Meier method and cox proportional hazard regression was applied to examine the factors associated with death. A p-value less than 0.05 was considered to indicate statistically significance.

## Supplementary information

Supplementary Figure and Table.

Reproducibility Checklist

Original Western Blots

## Data Availability

The raw data supporting the conclusions of this article will be made available by the authors on request.
