# Supplementary material for: Deficiency of LIMA1 accelerates bladder cancer progression by disrupting PINK1/Parkin-mediated mitophagy
Source: Cell Death Discov. 2026 Jun 16;12:345. doi: 10.1038/s41420-026-03136-5 (PMC13507292; doi:10.1038/s41420-026-03136-5)
Supplement: Supplementary file 1 [file 41420_2026_3136_MOESM2_ESM.pdf]

SPRINGER NATURE

Corresponding Author Name: Zhong Lv
Manuscript Number: CDDISCOVER

Reporting Summary

Springer Nature wishes to improve the reproducibility of the work that we publish. This checklist is used to ensure good reporting standards and to improve the reproducibility. Please respond completely to all questions relevant to your manuscript. For more information, please read the journal's Guide to Authors.

☑ **Check here to confirm that the following information is available in the Material & Methods section:**

- **The exact sample size (*n*)** for each experimental group/condition, given as a number, not a range
- **A description of the sample collection** allowing the reader to understand whether the samples represent technical or biological replicates (including how many animals, litters, culture, etc.)
- **A statement of how many times the experiment shown was replicated in the laboratory**
- **Definitions of statistical methods and measures**: For small sample sizes (n<5) descriptive statistics are not appropriate, instead plot individual data points
    - Very common tests, such as *t*-test, simple χ^2 tests, Wilcoxon and Mann-Whitney tests, can be unambiguously identified by name only, but more complex techniques should be described in the methods section
    - Are tests one-sided or two-sided?
    - Are there adjustments for multiple comparisons?
    - **Statistical test results**, e.g., ***P* values**
    - Definition of **'center values'** as **median or mean**;
    - Definition of **error bars as s.d. or s.e.m. or c.i.**

Please ensure that the answers to the following questions are reported in the manuscript itself. We encourage you to include a specific subsection in the methods section for statistics, reagents and animal models. Below, provide the page number or section and paragraph number.

Statistics and general methods

	Reported in section/paragraph or page #
1. How was the sample size chosen to ensure adequate power to detect a pre-specified effect size? (Give section/paragraph or page #)	Section 2.1 and 2.19.<br>The sample sizes for both clinical specimens and animal experiments were determined based on a combination of preliminary experimental data and published literature in
For animal studies, include a statement about sample size estimate even if no statistical methods were used.	Section 2.18.<br>Sample sizes (n=6 per group) were estimated based on previous literature and preliminary data to ensure sufficient power to detect significant differences in tumor growth
2. Describe inclusion/exclusion criteria if samples or animals were excluded from the analysis. Were the criteria pre-established? (Give section/paragraph or page #)	Section 2.1 and 2.18.<br>The inclusion and exclusion criteria for clinical spec
3. If a method of randomization was used to determine how samples/animals were allocated to experimental groups and processed, describe it. (Give section/paragraph or page #)	Section 2.1 and 2.18.<br>For clinical specimens (Section 2.1), samples were grouped based on pre-confirmed pathological diagnoses (BLCA vs. Healthy), and laboratory processing of these samples (ELISA, IHC, etc.) was conducted in a randomized sequence
For animal studies, include a statement about randomization even if no randomization was used.	Randomization was implemented during the allocation of mice to experimental groups. The mice were randomly assigned to either the negative control vector group or the

4. If the investigator was blinded to the group allocation during the experiment and/or when assessing the outcome, state the extent of blinding. (Give section/paragraph or page #)	Section 2.5.<br>Blinding was implemented during the evaluation of experimental outcomes to ensure objectivity. As reported in Section 2.5, the scoring of immunohistochemical (IHC) staining (intensity and frequency) was conducted in a
For animal studies, include a statement about blinding even if no blinding was done.	The measurement of tumor volumes and weights in the animal model was performed by researchers who were blinded to the group allocations during the assessment
5. For every figure, are statistical tests justified as appropriate?	Section 2.19.<br>Yes, statistical tests for every figure were justified based on data distribution and experimental design. As detailed in
Do the data meet the assumptions of the tests (e.g., normal distribution)?	Yes, all data were rigorously tested to ensure they met the assumptions of the specific statistical tests applied. As stated in Section 2.19, the Shapiro-Wilk test was used to
Is there an estimate of variation within each group of data?	Yes, an estimate of variation is provided for all quar
Is the variance similar between the groups that are being statistically compared? (Give section/paragraph or page #)	Yes, the variance was evaluated to ensure it was similar between compared groups. Homogeneity of variance was assessed before proceeding with parametric tests like t-tests or ANOVA. In cases where the assumption of equal

Reagents

Reported in section/paragraph or page #

6. Report the source of antibodies (vendor and catalog number)	Section 2.3 and 2.5.<br>All antibodies used in this study, including their specific vendors (suppliers), catalog numbers, and application
7. Identify the source of cell lines and report if they were recently authenticated (e.g., by STR profiling) and tested for mycoplasma contamination	Section 2.6.<br>All cell lines used in this study (TCCSUP, T24, 5637, and SVHUC-1) were purchased from Vigen Biotechnology Co., Ltd. As stated in Section 2.6, the authenticity of these cell lines was recently verified using short tandem repeat (STR)

Animal Models

Reported in section/paragraph or page #

8. Report species, strain, sex and age of animals	Section 2.18.<br>We used 12 NOD-Prkdcscid Il2rgtm201(-/-)/V high
9. For experiments involving live vertebrates, include a statement of compliance with ethical regulations and identify the committee(s) approving the experiments.	Section 2.18.<br>All animal experimental procedures were approved by the Institutional Animal Management & Use Committee of Jiangsu University (approval number: UJS-IACUC-AP-2024022801)

10. We recommend consulting the ARRIVE guidelines (*PLoS Biol.* **8**(6), e1000412,2010) to ensure that other relevant aspects of animal studies are adequately reported.

Human subjects

	Reported in section/paragraph or page #
11. Identify the committee(s) approving the study protocol.	Section 2.1.<br>Approved by the Institutional Ethics Committee of Wujin<br>Hospital of Public Jiangsu University (Approval Number:
12. Include a statement confirming that informed consent was obtained from all subjects.	Section 2.1.<br>I confirm that written informed consent was obtained from all<br>participants involved in this study. All subjects provided their
13. For publication of patient photos, include a statement confirming that consent to publish was obtained.	Not applicable.
14. Report the clinical trial registration number (at ClinicalTrials.gov or equivalent).	Not applicable.

15. For phase II and III randomized controlled trials, please refer to the CONSORT statement and submit the CONSORT checklist with your submission.

16. For tumor marker prognostic studies, we recommend that you follow the REMARK reporting guidelines.

Data deposition

	Reported in section/paragraph or page #
17. Provide accession codes for deposited data. Data deposition in a public repository is mandatory for:<br>a. Protein, DNA and RNA sequences<br>b. Macromolecular structures<br>c. Crystallographic data for small molecules<br>d. Microarray data	Not applicable.

Deposition is strongly recommended for many other datasets for which structured public repositories exist; more details on our data policy are available in the Guide to Authors. We encourage the provision of other source data in supplementary information or in unstructured repositories such as Figshare and Dryad. We encourage publication of Data Descriptors (see Scientific Data) to maximize data reuse.

18. If computer code was used to generate results that are central to the paper's conclusions, include a statement in the Methods section under "**Code availability**" to indicate whether and how the code can be accessed. Include version information as necessary and any restrictions on availability.	Not applicable.
