# Supplementary material for: Deficiency of LIMA1 accelerates bladder cancer progression by disrupting PINK1/Parkin-mediated mitophagy
Source: Cell Death Discov. 2026 Jun 16;12:345. doi: 10.1038/s41420-026-03136-5 (PMC13507292; doi:10.1038/s41420-026-03136-5)

Uncropped blot images of western blots shown in Fig. 2A

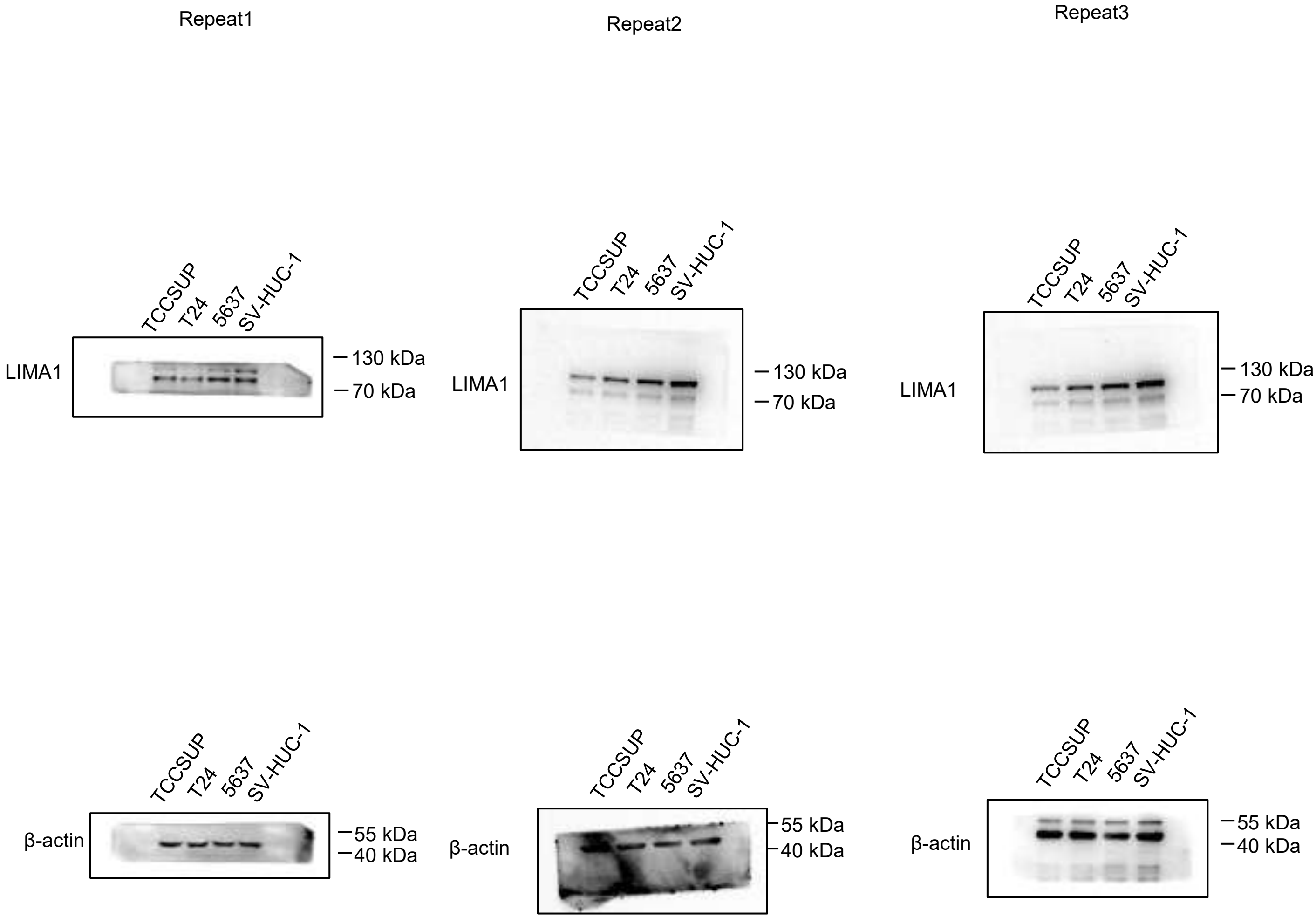

Uncropped blot images of western blots shown in Fig. 2D

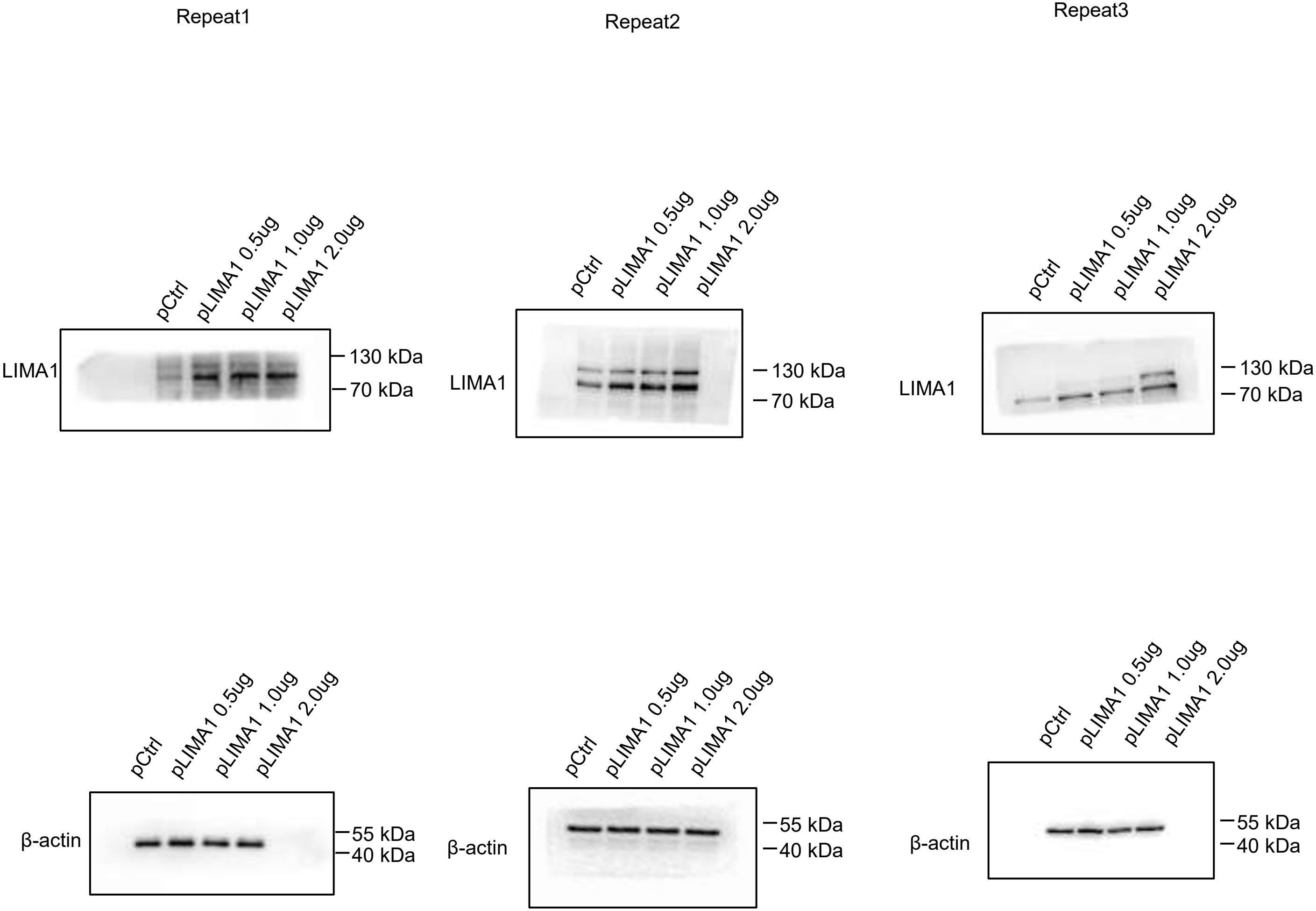

Uncropped blot images of western blots shown in Fig. 2E

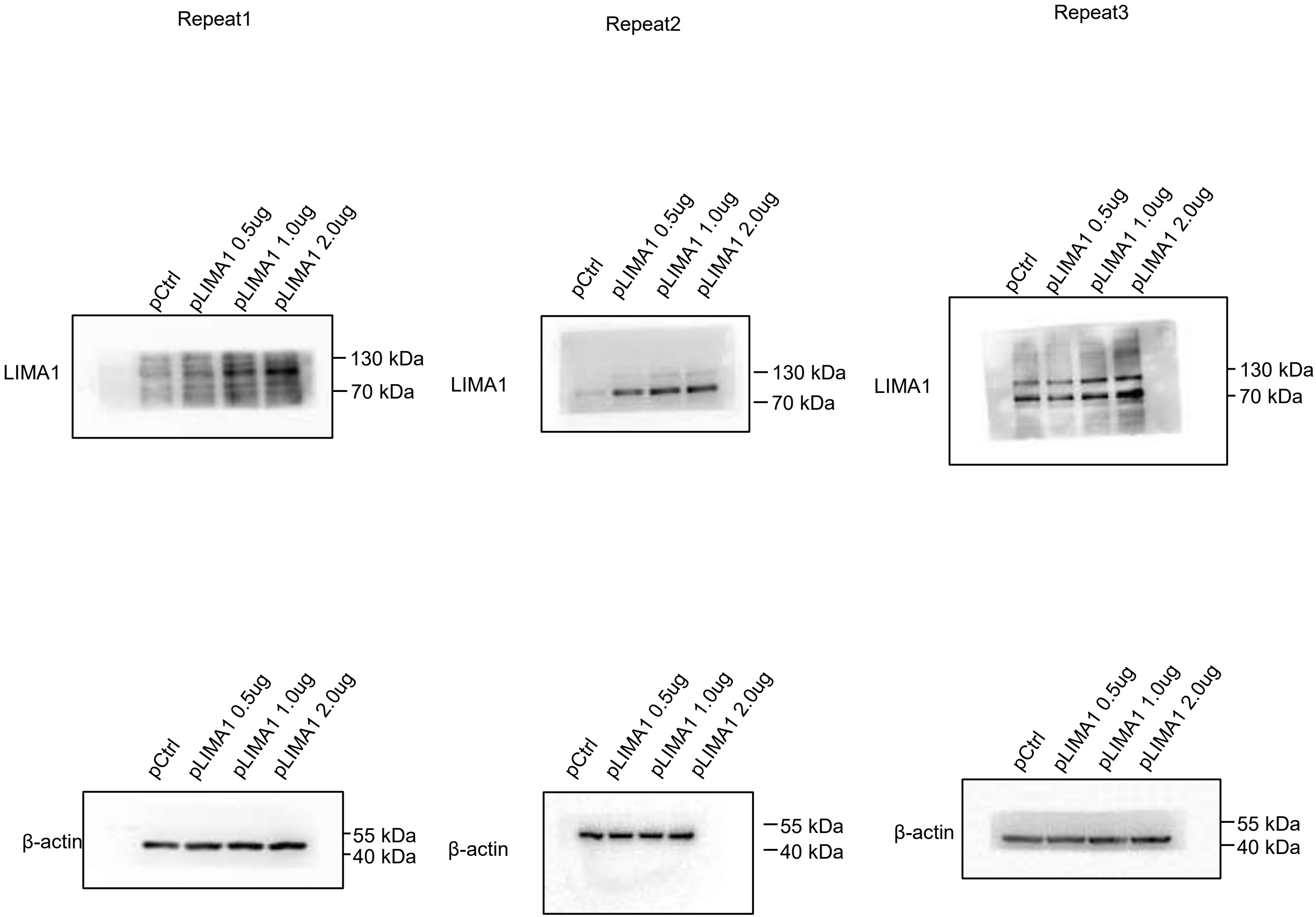

Uncropped blot images of western blots shown in Fig. 3A

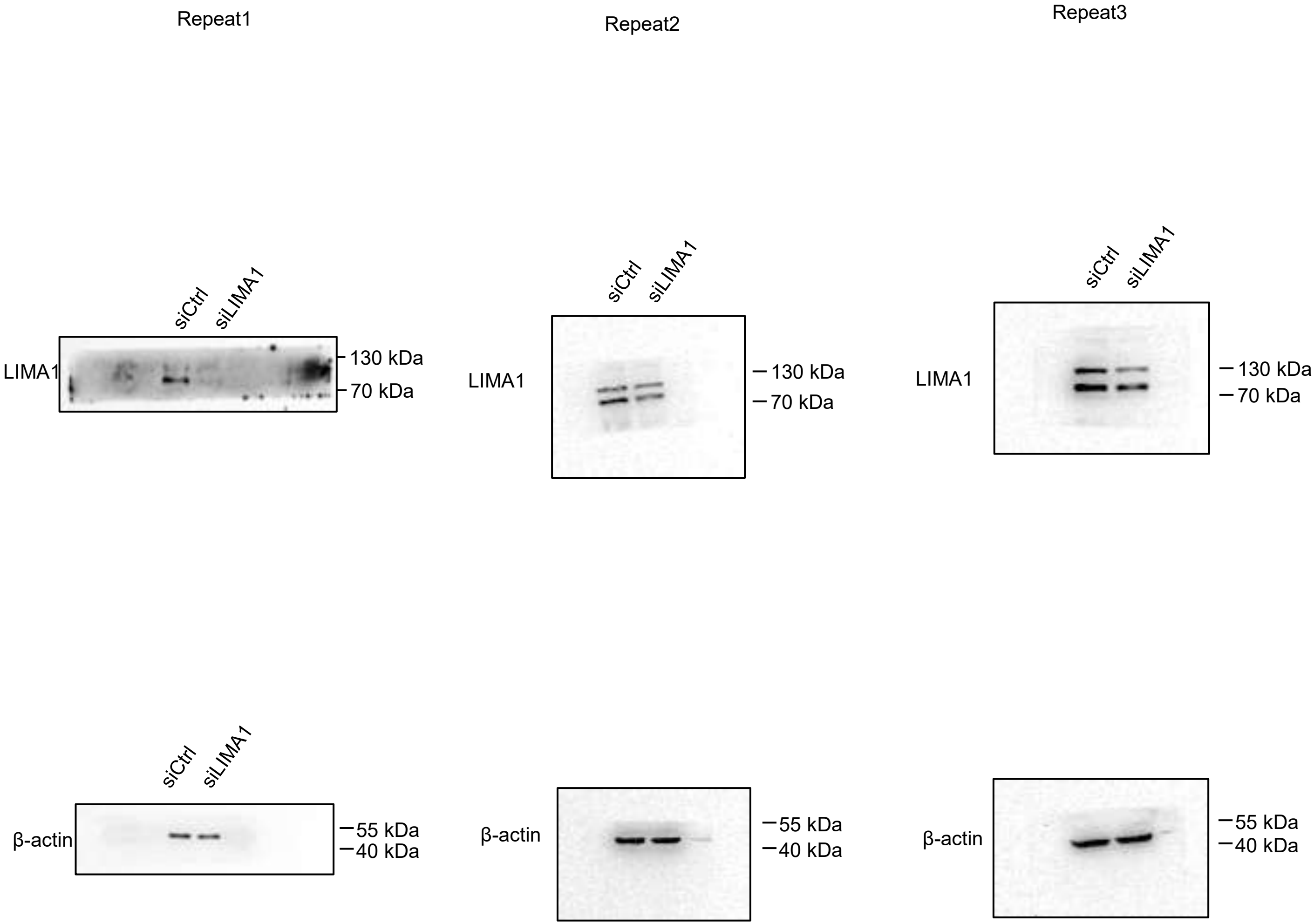

Uncropped blot images of western blots shown in Fig. 3C

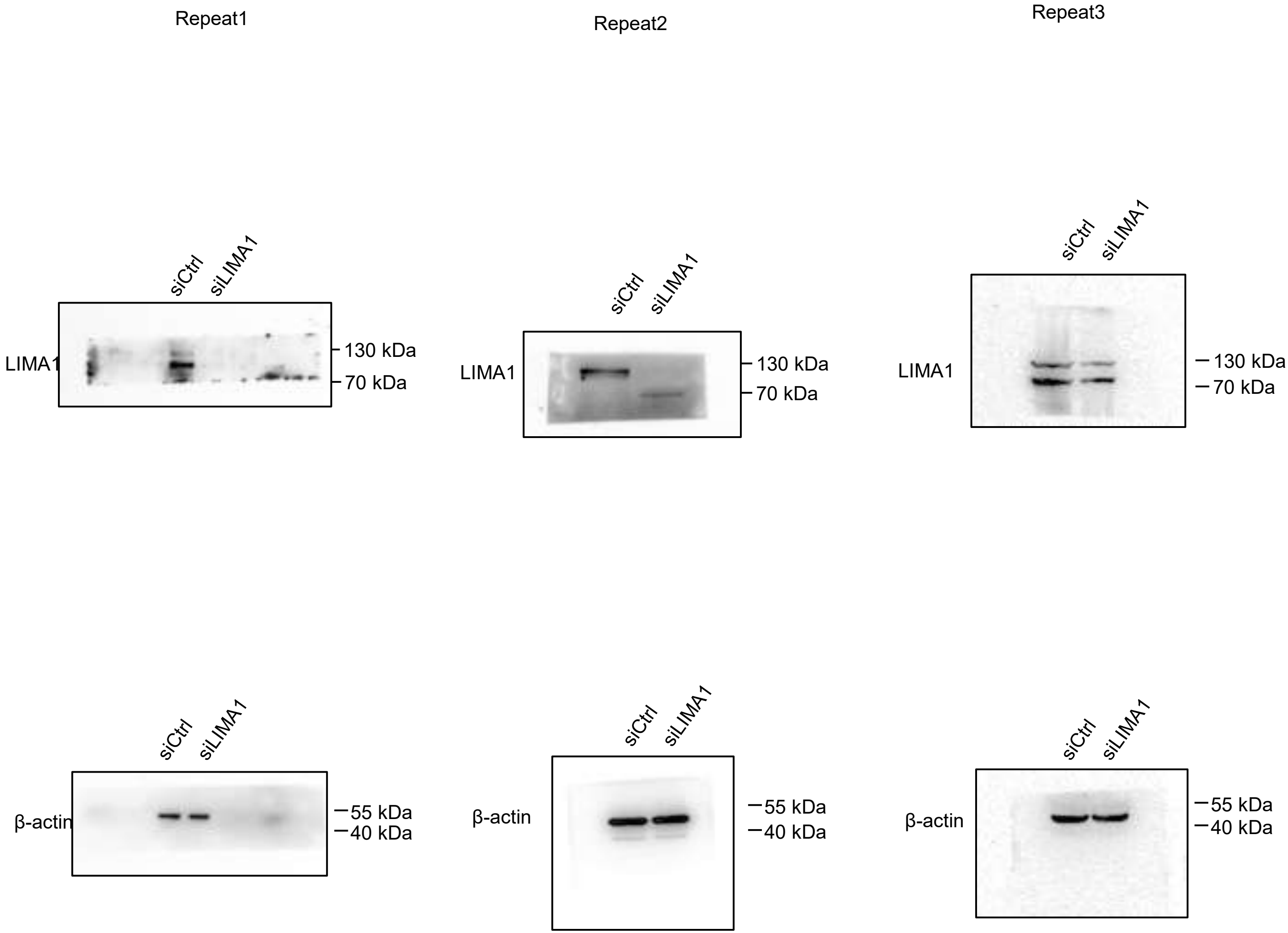

Uncropped blot images of western blots shown in Fig. 7B

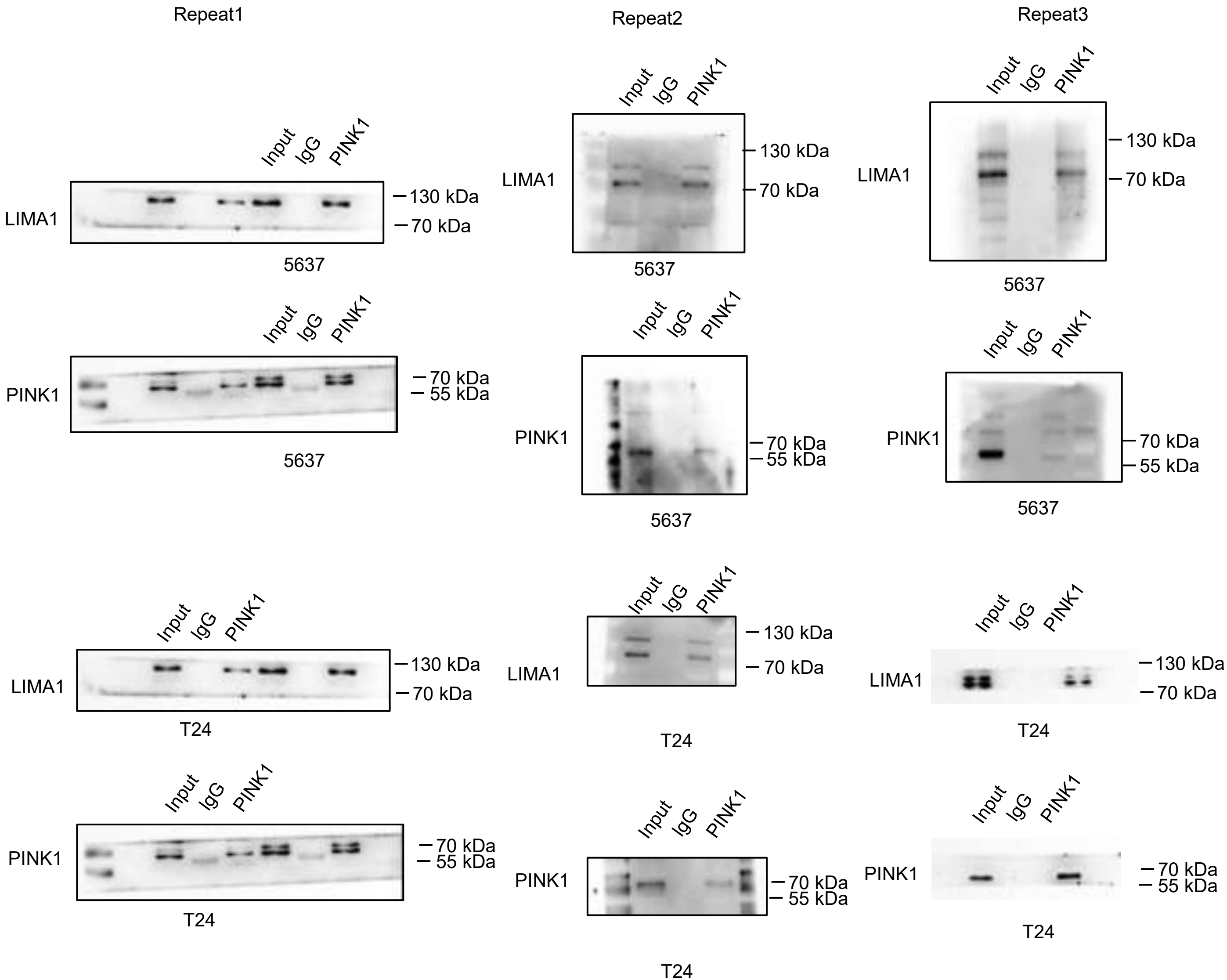

Uncropped blot images of western blots shown in Fig. 7D

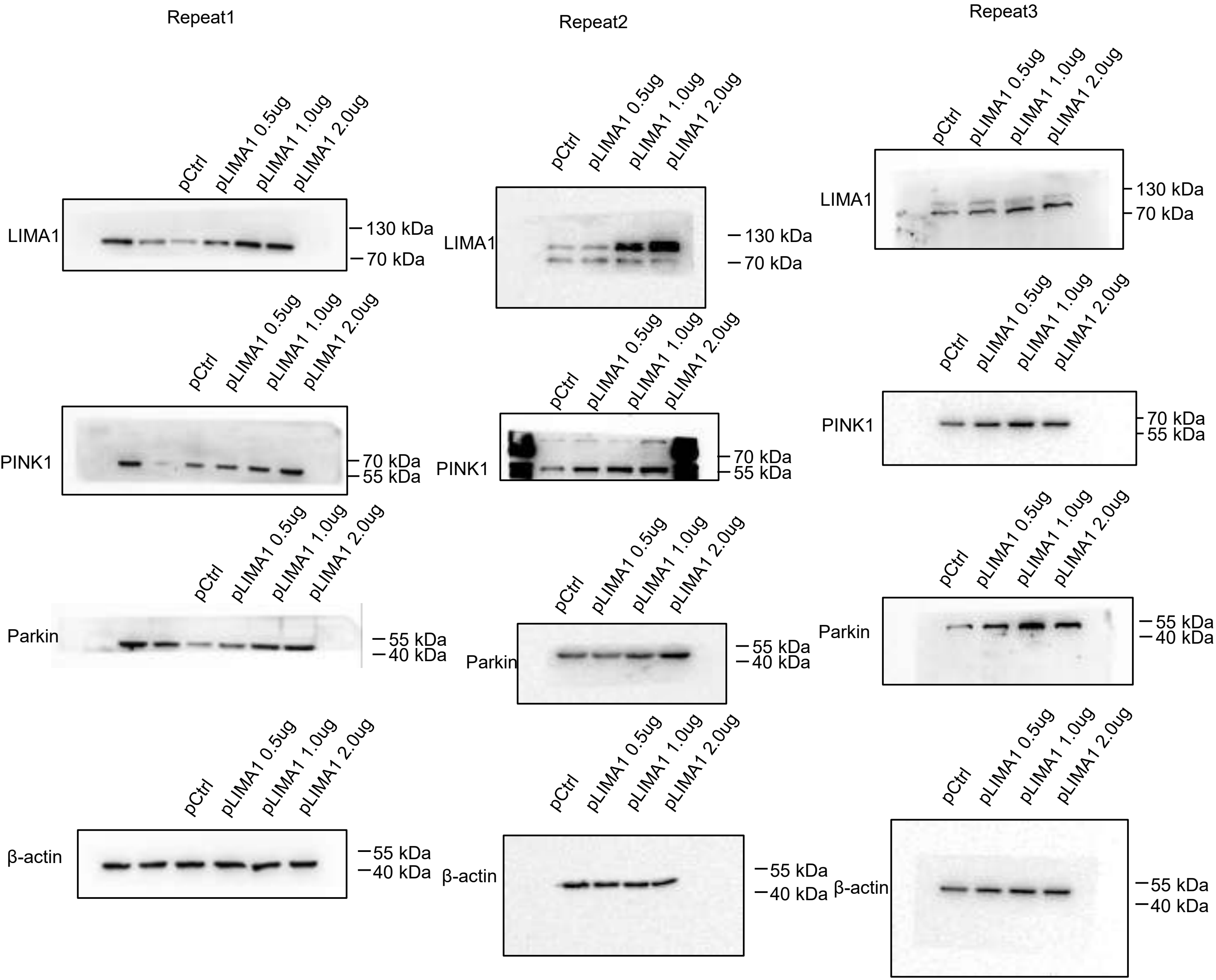

Uncropped blot images of western blots shown in Fig. 7F

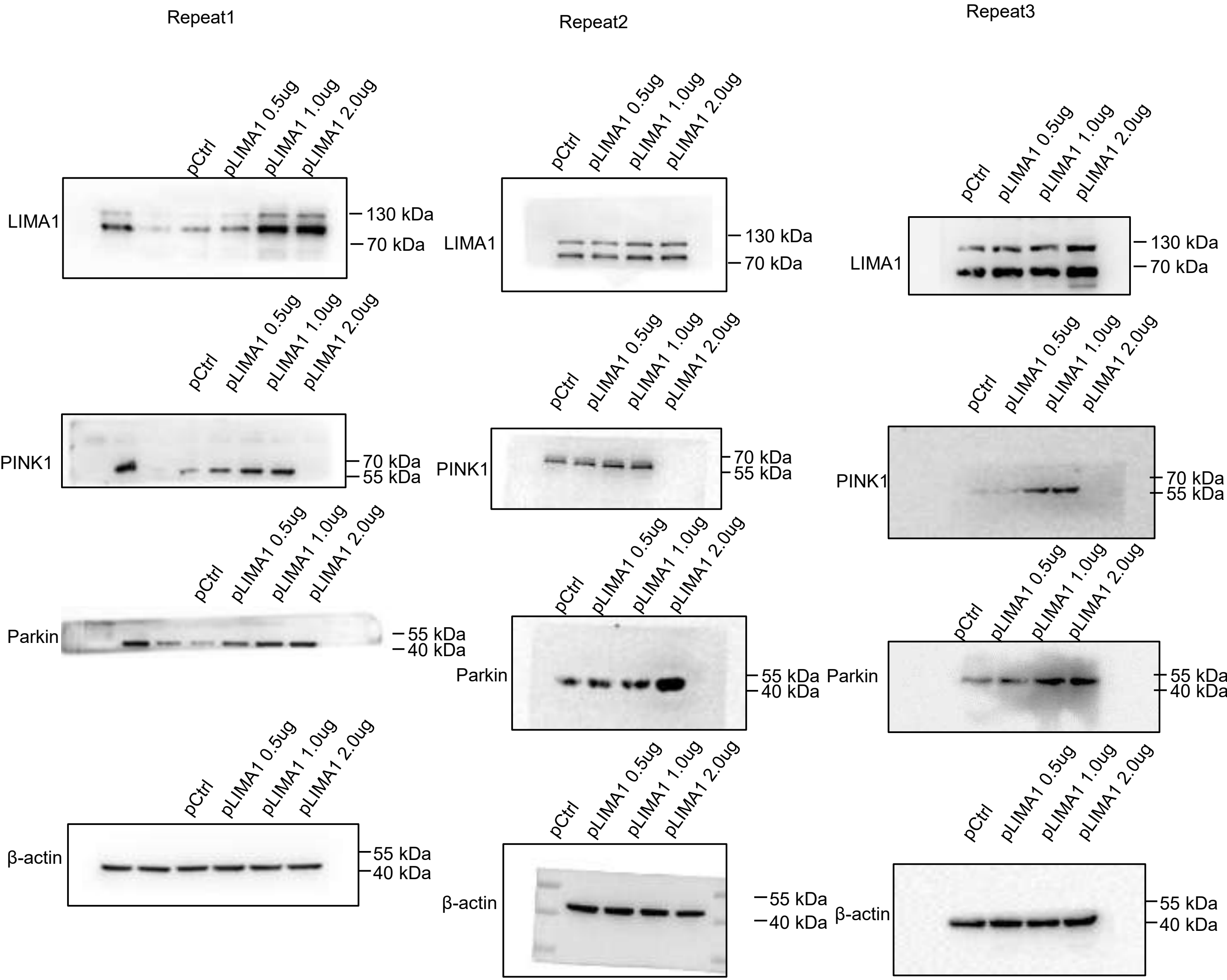

Uncropped blot images of western blots shown in Fig. 7H

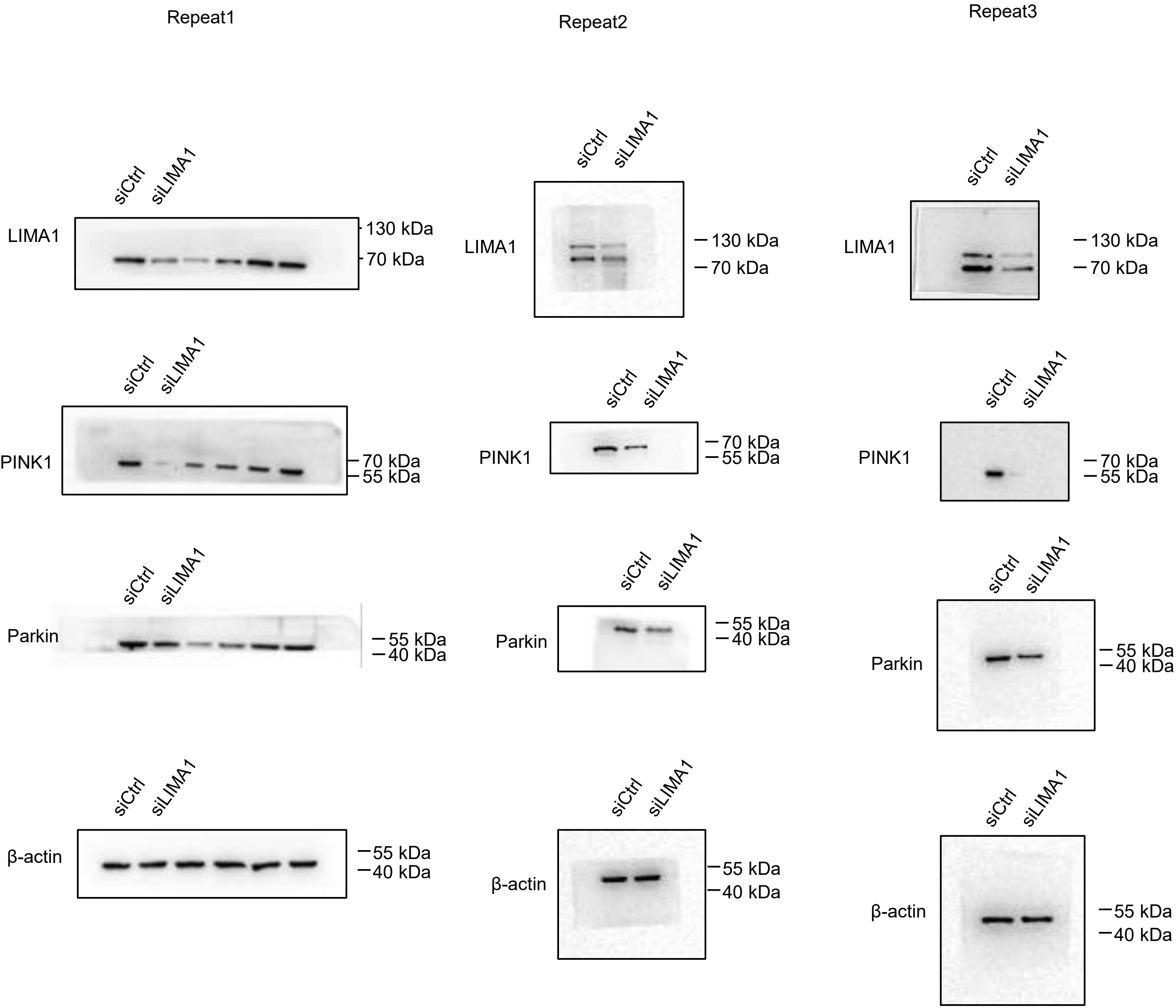

Uncropped blot images of western blots shown in Fig. 7J

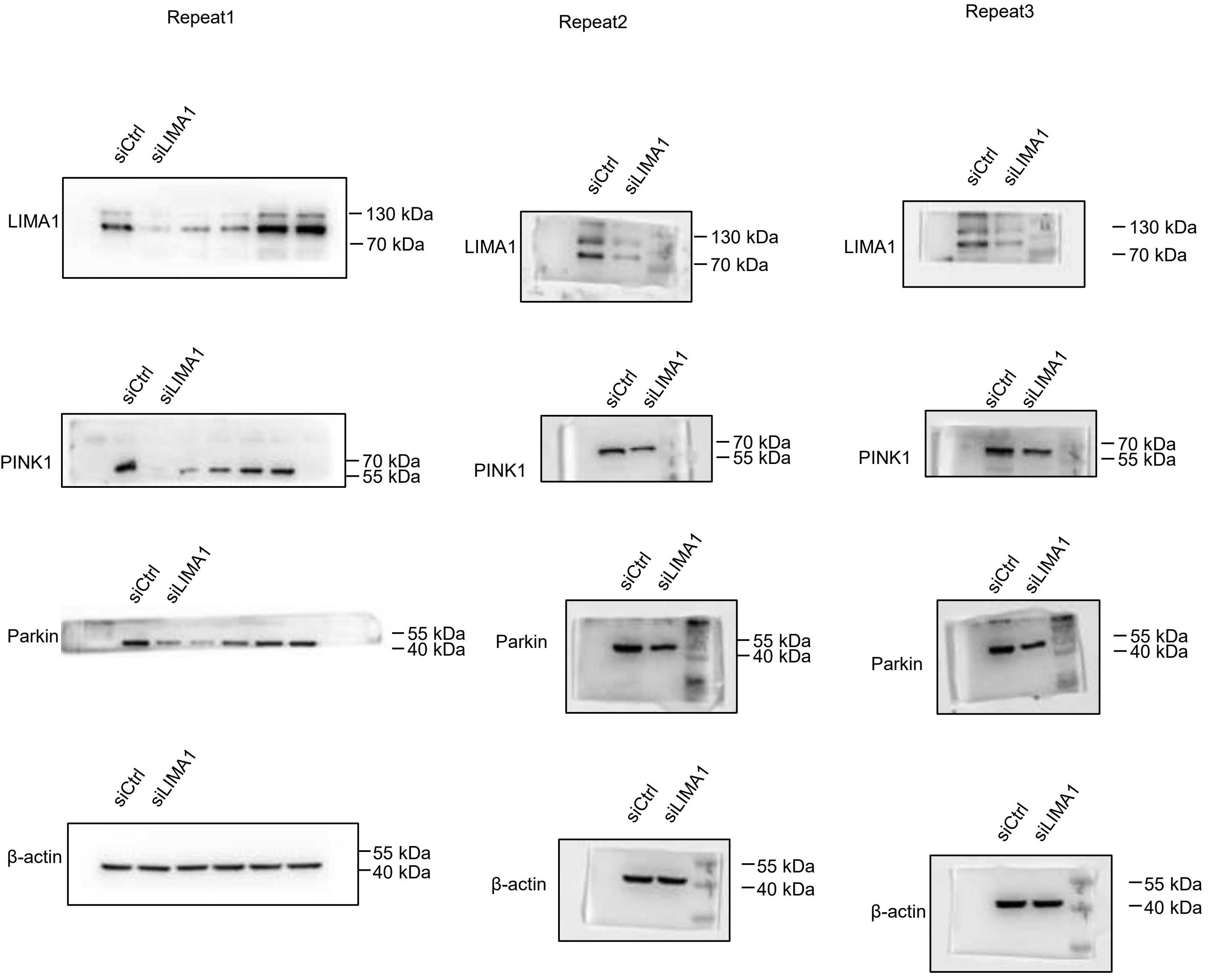

Supplement: Supplementary file 2 [file 41420_2026_3136_MOESM3_ESM.pdf]
