# Supplementary material for: Deficiency of LIMA1 accelerates bladder cancer progression by disrupting PINK1/Parkin-mediated mitophagy
Source: Cell Death Discov. 2026 Jun 16;12:345. doi: 10.1038/s41420-026-03136-5 (PMC13507292; doi:10.1038/s41420-026-03136-5)
Supplement: Supplementary file 3 [file 41420_2026_3136_MOESM1_ESM.docx]

**Supplementary Figure 1.** The expression level of LIMA1 differed according to clinical stage or pathologic stage.

**A.** The relationship between LIMA1 concentration of serum and clinical stage was determined by using the Kruskal-Wallis test. **B.** The relationship between LIMA1 concentration of serum and pathologic stage was determined by using the Kruskal-Wallis test. **C.** The relationship between the staining score of LIMA1 and clinical stage was determined by using the Kruskal-Wallis test. **D.** The relationship between the staining score of LIMA1 and pathologic stage was determined by using the Kruskal-Wallis test. **P* < 0.05; ***P* < 0.01.

**Supplementary Figure 2.** Discrimination of LIMA1 expression in serum and establishment of a predictive diagnostic panel.

**A.** ROC curve analysis of serum LIMA1 expression in distinguishing BLCA patients from healthy controls. *****P* < 0.0001. **B.** ROC curve analysis of serum LIMA1 expression in distinguishing clinical stage 0a- Ⅱ from Ⅲ- Ⅳ in BLCA patients. ***P* = 0.0012. **C.** ROC curve analysis of serum LIMA1 expression in distinguishing T stage T_a_- T_2_ from T_3_- T_4_ in BLCA patients. ***P* = 0.0012. **D.** ROC curve analysis of serum LIMA1 expression in distinguishing N stage N_0_ from N_x_ in BLCA patients. ****P* = 0.0002. **E.** ROC curve analysis of serum LIMA1 expression in distinguishing pathologic stage Ⅰ- Ⅱ from Ⅲ in BLCA patients. **P* = 0.0161. **F.** ROC curve analysis of serum LIMA1 expression in distinguishing stable group from relapse group in BLCA patients. **P* = 0.017.

**Supplementary Figure 3.** Discrimination of LIMA1 expression in histology and establishment of a predictive diagnostic panel.

**A, B.** ROC curve analysis of tissue LIMA1 expression in distinguishing BLCA patients from healthy controls. *****P* < 0.0001 **(A)**, ****P* =0.0005 **(B)**. **C.** ROC curve analysis of tissue LIMA1 expression in distinguishing clinical stage 0a- Ⅱ from Ⅲ- Ⅳ in BLCA patients. ***P* =0.009. **D.** ROC curve analysis of tissue LIMA1 expression in distinguishing T stage T_a_- T_2_ from T_3_- T_4_ in BLCA patients. **P* =0.0292. **E.** ROC curve analysis of serum LIMA1 expression in distinguishing N stage N_0_ from N_x_ in BLCA patients. ***P* =0.0072. **F.** ROC curve analysis of serum LIMA1 expression in distinguishing pathologic stage Ⅰ- Ⅱ from Ⅲ in BLCA patients. *P* =0.09.

**Supplementary Table 1.** Summary of primary antibodies used in Western Blot, Immunochemistry, immunofluorescence and Co-immunoprecipitation.

| Concentration | | | | | | |
| --- | --- | --- | --- | --- | --- | --- |
| Antibody | for WB | for IHC | for IF | for co-IP | Cat. No. | Company |
| LIMA1 | 1:1000 | 1:100 | 1:100 | 1:50 | 66071-1-lg | Proteintech |
| Ki67 |  | 1:600 |  |  | GB111499 | Proteintech |
| PINK1 | 1:1000 | 1:50 | / | 1:50 | A7131 | Abclonal |
| Parkin | 1:1000 | / | / | / | A0968 | Abclonal |
| β-actin | 1:5000 | / | / | / | AC006 | Abclonal |
| TOM20 | / | / | 1:100 | / | 11802-1-AP | Proteintech |
| LC3B | / | / | 1:100 | / | A17424 | Abclonal |
| HRP Goat Anti-Rabbit IgG (H+L) | 1:5000 | / | / | / | AS014 | Abclonal |
| HRP Goat Anti-Mouse IgG (H+L) | 1:5000 | / | / | / | AS003 | Abclonal |
| Mouse Anti-Rabbit IgG LCS | / | / | / | 1:2000 | A25022 | IPKine |
| Goat Anti-Rabbit IgG HCS | / | / | / | 1:2000 | A25222 | IPKine |
| Cy3-Goat Anti-Mouse IgG (H+L) | / | 1:200 | / | / | AS008 | Abclonal |
| FITC-Goat Anti-Rabbit IgG (H+L) | / | 1:200 | / | / | AS011 | Abclonal |
| Cy3 Goat Anti-Rabbit IgG (H+L) | / | 1:200 | / | / | AS007 | Abclonal |
| FITC Goat Anti-Mouse IgG (H+L) | / | 1:200 | / | / | AS001 | Abclonal |

Abbreviation: Co-IP, immunoprecipitation; WB, western blot; IHC, immunohistochemistry; IF, immunofluorescence.

**Supplementary Table 2.** Primers in this study.

| Identifier | Forward (5’-3’) | Reverse (5’-3’) |
| --- | --- | --- |
| **For qPCR** | | |
| LIMA1 | CCAAGCCACTAAGTCCAGATTCCAG | GACATTCCACGCAGGTCTCTCTTG |
| PINK1 | GAAGCCAUCUUGAACACAATT | UUGUGUUCAAGAUGGCUUCTT |
| Parkin | GTGCAGAGACCGTGGAGAAA | GCTGCACTGTACCCTGAGTT |
| β-actin | GGCCAACCGCGAGAAGATGAC | GGATAGCACAGCCTGGATAGCAAC |

Abbreviation: qPCR, quantitative real-time polymerase chain reaction.

**Supplementary Table 3.** RNA oligo in this study.

| Identifier | Sense | Antisense |
| --- | --- | --- |
| **For Knockdown** | | |
| LIMA1 oligo | GCCCAAAGCCGAAGUGCAATT | UUGCACUUCGGCUUUGGGCTT |
| Control oligo | UUCUCCGAACGUGUCACGUTT | ACGUGACACGUUCGGAGAATT |

| Characteristics | LIMA1 expression in serum | | Chi-square | *p* value |
| --- | --- | --- | --- | --- |
|  | Low | High |  |  |
| Age |  |  | 0.1968 | 0.6573 |
| ≤65 | 12 | 10 |  |  |
| ＞65 | 14 | 15 |  |  |
| Gender |  |  | 2.407 | 0.1208 |
| Male | 11 | 16 |  |  |
| Female | 15 | 9 |  |  |
| T stage |  |  | 8.816 | 0.0030 |
| Ta-T_2_ | 9 | 19 |  |  |
| T_3_-T_4_ | 17 | 6 |  |  |
| N stage |  |  | 7.994 | 0.0047 |
| N_0_ | 12 | 21 |  |  |
| N_X_ | 14 | 4 |  |  |
| M stage |  |  | 7.802 | 0.0052 |
| M_0_ | 19 | 25 |  |  |
| M_1_ | 7 | 0 |  |  |
| Clinical stage |  |  | 8.816 | 0.0030 |
| 0a-Ⅱ | 9 | 19 |  |  |
| Ⅲ-Ⅳ | 17 | 6 |  |  |
| Pathologic stage |  |  | 6.744 | 0.0094 |
| Ⅰ-Ⅱ | 3 | 11 |  |  |
| Ⅲ | 23 | 14 |  |  |

**Supplementary Table 4.** Clinical characteristics of BLCA (n=51).

Note: The median of LIMA1 expression in serum was used to distinguish between low (n=26) and high (n=25) groups.

**Supplementary Table 5.** Summarized diagnostic factors of the serum LIMA1 expression.

| Diagnostic factors | | Serum concentrations of LIMA1 (mean, SD) | Cut-off Value | AUC (95%CI) | Sensitivity (%) | Specificity (%) |
| --- | --- | --- | --- | --- | --- | --- |
| Subjects | BLCA | 1.39(0.90) | >3.04 | 0.93(0.87-0.99) | 88.57 | 94.12 |
|  | NC | 3.94(1.30) |  |  |  |  |
| Clinical stage | 0a- Ⅱ | 1.70(0.93) | <0.80 | 0.77(0.63-0.90) | 56.52 | 92.86 |
|  | Ⅲ-Ⅳ | 0.93(0.65) |  |  |  |  |
| T stage | Ta-T_2_ | 1.70(0.93) | <0.80 | 0.77(0.63-0.90) | 56.52 | 92.86 |
|  | T_3_-T_4_ | 0.93(1.00) |  |  |  |  |
| N stage | N_0_ | 1.69(0.93) | <0.80 | 0.82(0.70-0.94) | 63.16 | 90.63 |
|  | N_X_ | 0.78(0.44) |  |  |  |  |
| Pathologic stage | Ⅰ-Ⅱ | 1.86(1.10) | <1.40 | 0.72(0.56-0.88) | 75.68 | 71.43 |
|  | Ⅲ | 1.16(0.73) |  |  |  |  |
| Concentration | stable | 1.79(0.94) | <0.71 | 0.74(0.57-0.91) | 46.15 | 100.00 |
|  | relapse | 0.99(1.06) |  |  |  |  |

Abbreviations: AUC, area under the curve; CI, confidence interval; ROC curve, receiver operating characteristic curve

**Supplementary Table 6.** Univariate Cox analysis.

|  | **B** | **SE** | **Wald** | **df** | **Sig.** | **Exp(B)** | **95% CI** | |
| --- | --- | --- | --- | --- | --- | --- | --- | --- |
|  |  |  |  |  |  |  | **down** | **up** |
| Age | 0.019 | 0.016 | 1.482 | 1 | 0.223 | 1.019 | 0.988 | 1.052 |
| Gender | 0.009 | 0.364 | 0.001 | 1 | 0.981 | 1.009 | 0.494 | 2.060 |
| T stage | 1.603 | 0.322 | 24.774 | 1 | 0.001 | 4.969 | 2.643 | 9.343 |
| N stage | 2.948 | 0.523 | 31.753 | 1 | 0.001 | 19.076 | 6.941 | 53.193 |
| M stage | 2.648 | 0.577 | 21.036 | 1 | 0.001 | 14.132 | 4.557 | 43.823 |
| Clinical stage | 2.222 | 0.391 | 32.349 | 1 | 0.001 | 9.228 | 4.291 | 19.846 |
| Pathological stage | 0.684 | 0.336 | 4.132 | 1 | 0.042 | 1.981 | 1.025 | 3.829 |
| LIMA1 | 1.225 | 0.432 | 8.057 | 1 | 0.005 | 3.405 | 1.461 | 7.936 |

Note: B: Beta coefficient, SE: Standard Error, Wald: Wald’s chi-square value, df: degree of freedom, Sig.: significance, Exp(B): The exponent of B, CI: confidence interval.

**Supplementary Table 7.** Clinical characteristics of BLCA (n=63).

| Characteristics | Staining score of LIMA1 | | Chi-square | *p* value |
| --- | --- | --- | --- | --- |
|  | Low | High |  |  |
| Age |  |  | 0.0002 | 0.9870 |
| ≤65 | 20 | 14 |  |  |
| ＞65 | 17 | 12 |  |  |
| Gender |  |  | 0.19650 | 0.6576 |
| Male | 22 | 14 |  |  |
| Female | 15 | 12 |  |  |
| T stage |  |  | 4.6360 | 0.0313 |
| Ta-T_2_ | 14 | 17 |  |  |
| T_3_-T_4_ | 23 | 9 |  |  |
| N stage |  |  | 8.3530 | 0.0039 |
| N_0_ | 27 | 26 |  |  |
| N_X_ | 10 | 0 |  |  |
| M stage |  |  | 1.0140 | 0.3140 |
| M_0_ | 33 | 25 |  |  |
| M_1_ | 4 | 1 |  |  |
| Clinical stage |  |  | 6.3090 | 0.0120 |
| 0a-Ⅱ | 11 | 16 |  |  |
| Ⅲ-Ⅳ | 26 | 10 |  |  |
| Pathologic stage |  |  | 0.1048 | 0.7462 |
| Ⅰ-Ⅱ | 10 | 8 |  |  |
| Ⅲ | 27 | 18 |  |  |

Note: The median of staining scores was used to distinguish between low (n=37) and high (n=26) groups.

**Supplementary Table 8.** Summarized diagnostic factors of the histology LIMA1 expression.

| Diagnostic factors | | The IHC score of LIMA1 (mean, SD) | Cut-off Value | AUC (95%CI) | Sensitivity (%) | Specificity (%) |
| --- | --- | --- | --- | --- | --- | --- |
| Subjects (staining positive intensity) | BLCA | 0.62(0.06) | >0.65 | 0.89(0.83-0.96) | 90.63 | 84.13 |
|  | NC | 0.70(0.05) |  |  |  |  |
| Subjects (staining score) | BLCA | 6.51(2.78) | >7.00 | 0.72(0.62-0.82) | 71.88 | 58.73 |
|  | NC | 8.59(2.35) |  |  |  |  |
| Clinical stage | 0a- Ⅱ | 7.74(3.02) | <10.50 | 0.69(0.56-0.82) | 100.00 | 25.93 |
|  | Ⅲ-Ⅳ | 5.58(2.20) |  |  |  |  |
| T stage | Ta-T_2_ | 7.47(3.06) | <10.50 | 0.66(0.53-0.79) | 100.00 | 23.33 |
|  | T_3_-T_4_ | 5.64(2.19) |  |  |  |  |
| N stage | Ta-T_2_ | 6.91(2.74) | <5.00 | 0.77(0.61-0.93) | 80.00 | 75.47 |
|  | T_3_-T_4_ | 4.40(1.96) |  |  |  |  |
| Pathologic stage | Ⅰ-Ⅱ | 7.82(2.35) | <5.00 | 0.64(0.50-0.78) | 32.61 | 94.12 |
|  | Ⅲ | 6.35(2.66) |  |  |  |  |

Abbreviations: IHC, Immunohistochemistry; AUC, area under the curve; CI, confidence interval; ROC curve, receiver operating characteristic curv

**Supplementary Table 9.** Multivariate Cox analysis.

|  | **B** | **SE** | **Wald** | **df** | **Sig.** | **Exp(B)** | **95% CI** | |
| --- | --- | --- | --- | --- | --- | --- | --- | --- |
|  |  |  |  |  |  |  | **down** | **up** |
| T stage | 0.634 | 0.509 | 1.550 | 1 | 0.213 | 1.884 | 0.695 | 5.109 |
| N stage | 0.608 | 0.919 | 0.437 | 1 | 0.509 | 1.836 | 0.303 | 11.130 |
| M stage | 0.899 | 0.842 | 1.142 | 1 | 0.285 | 2.458 | 0.472 | 12.794 |
| Clinical stage | 1.648 | 0.682 | 5.840 | 1 | 0.016 | 5.194 | 1.365 | 19.762 |
| Pathological stage | 0.893 | 0.409 | 4.778 | 1 | 0.029 | 2.444 | 1.097 | 5.444 |
| LIMA1 | 1.407 | 0.491 | 8.222 | 1 | 0.004 | 4.083 | 1.561 | 10.681 |

Note: B: Beta coefficient, SE: Standard Error, Wald: Wald’s chi-square value, df: degree of freedom, Sig.: significance, Exp(B): The exponent of B, CI: confidence interval.
